# Unlocking the potential of senescence-related gene signature as a diagnostic and prognostic biomarker in sepsis: insights from meta-analyses, single-cell RNA sequencing, and *in vitro* experiments

**DOI:** 10.18632/aging.205574

**Published:** 2024-02-26

**Authors:** Jia Chen, Jinhong Si, Qiankun Li, Weihong Zhang, Jiahao He

**Affiliations:** 1 Department of Emergency, Panyu Maternal and Child Care Service Centre of Guangzhou, Hexian Memorial Affiliated Hospital of Southern Medical University, Panyu, Guangzhou 511400, Guangdong Province, China; 2 Department of Respiratory Medicine, Panyu Maternal and Child Care Service Centre of Guangzhou, Hexian Memorial Affiliated Hospital of Southern Medical University, Panyu, Guangzhou 511400, Guangdong Province, China

**Keywords:** sepsis, senescence, prognosis, diagnosis, immunity

## Abstract

Cellular senescence is closely associated with the pathogenesis of sepsis. However, the diagnostic and prognostic value of senescence-related genes remain unclear. In this study, 866 senescence-related genes were collected from CellAge. The training cohort, GSE65682, which included 42 control and 760 sepsis samples, was obtained from the Gene Expression Omnibus (GEO). Feature selection was performed using gene expression difference detection, LASSO analysis, random forest, and Cox regression. TGFBI and MAD1L1 were ultimately selected for inclusion in the multivariate Cox regression model. Clustering based on the expressions of TGFBI and MAD1L1 was significantly associated with sepsis characteristics and prognoses (all P < 0.05). The risk signature served as a reliable prognostic predictor across the GSE65682, GSE95233, and GSE4607 cohorts (pooled hazard ratio = 4.27; 95% confidence interval [CI] = 1.63-11.17). Furthermore, it also served as a robust classifier to distinguish sepsis samples from control cases across 14 cohorts (pooled odds ratio = 5.88; 95% CI = 3.54-9.77). Single-cell RNA sequencing analyses from five healthy controls and four sepsis subjects indicated that the risk signature could reflect the senescence statuses of monocytes and B cells; this finding was then experimentally validated in THP-1 and IM-9 cells *in vitro* (both P < 0.05). In all, a senescence-related gene signature was developed as a prognostic and diagnostic biomarker for sepsis, providing cut-in points to uncover underlying mechanisms and a promising clinical tool to support precision medicine.

## INTRODUCTION

Sepsis is a serious condition that can be life-threatening and is caused by an overactive immune response to infection, making it one of the leading causes of mortality in patients with severe infections [1]. In China, sepsis-related mortality rates are approximately 66.7 deaths per 100,000 population [2]. Globally, sepsis-related mortality accounts for 19.7% of all deaths [3]. Unfortunately, the lack of reliable and robust diagnostic and prognostic approaches is a major contributor to unfavorable clinical outcomes [4]. However, recent advancements in high-throughput sequencing and big-data analyses methods, including machine learning, offer potential for identifying novel biomarkers for diagnosis and prognosis prediction [5]. Despite these advances, no diagnostic biomarkers have yet been proven effective in clinical practice [6]. Therefore, identifying reliable and robust diagnostic and prognostic biomarkers remains the primary challenge lying ahead in sepsis research and the focus of this study.

Cellular senescence refers to the irreversible cessation of the cell cycle accompanied by impaired mitochondria metabolism, which is often induced by DNA injury, telomere shortening, and oxidative stress [7, 8]. Senescent cells undergo changes in gene expression and secrete a variety of bioactive molecules collectively called the senescence-associated secretory phenotype (SASP), which plays a pro-inflammatory role [9]. Given that inflammation serves as a crucial mechanism in sepsis initiation and progression, it is not surprising that a strong association between aging and the morbidity and mortality of sepsis has been observed by multiple epidemiological studies [10, 11]. Recent studies have also proven that viruses such as endogenous retroviruses [12] and severe acute respiratory syndrome coronavirus 2 [13] can elicit cellular senescence. All these pieces of evidence suggest that cellular senescence is tightly correlated with the pathogenesis of sepsis. Nevertheless, our understanding of their latent biological mechanisms is still limited.

In this study, we used multiple independent cohorts, single-cell RNA sequencing (scRNA-seq) data from 5 healthy control and 4 sepsis samples, and *in vitro* cellular experiments to identify senescence-related genes as potential diagnosis and prognosis biomarkers for sepsis. We employed machine learning-based algorithms such as least absolute shrinkage and selection operator (LASSO) regression, random forest, and Cox regression to perform feature selection and construct a risk model. The prognostic value of the model was validated in three large-scale independent datasets, while the diagnostic value of the model was confirmed in 14 datasets. We utilized scRNA-seq data to investigate the underlying mechanisms of these genes and confirmed our findings with cellular experiments conducted on THP-1 and IM-9 cells.

## MATERIALS AND METHODS

### Data collection and processing

We obtained 866 cellular senescence regulatory genes from the CellAge database (https://genomics.senescence.info/cells/) and have listed them in Supplementary Table 1. The training cohort, GSE65682 [14], consisted of transcriptome sequencing data from whole blood samples isolated from 42 healthy control and 760 sepsis subjects, along with their corresponding follow-up duration and survival statuses. We downloaded this dataset using the Gene Expression Omnibus (GEO, https://www.ncbi.nlm.nih.gov/geo/) and performed a comprehensive query of the GEO database using “sepsis” as the keyword, based on sample size in both control and sepsis groups being at least ten in “whole blood” type samples where transcriptome sequencing matrices included genes in the predictive model. To ensure accuracy, JS and QL independently performed manual queries using these criteria. Any discrepancies were resolved through discussion involving JC. Based on the search results, GSE4607 [15] and GSE95233 [16] datasets, which included follow-up information, were adopted to verify the prognostic value of the predictive model. Additionally, another 11 sepsis-related datasets (GSE9692 [17], GSE13904 [18], GSE26378 [19], GSE26440 [20], GSE28750 [21], GSE54514 [22], GSE57065 [23], GSE67652 [24], GSE69063, GSE69528 [25], and GSE131761 [26]) were selected to confirm the diagnostic value of the model. The sva package in R was used to minimize batch effects across these cohorts where possible.

To investigate the underlying mechanisms at a higher resolution, we downloaded GSE175453 [27] from the GEO, which contained scRNA-seq data from whole blood samples collected from 5 healthy control and 4 sepsis donors. The scRNA-seq data were processed using Seurat, which included data loading, quality control, and dimension reduction. Cell type annotation was performed using the SingleR package. Supplementary Material 1 contains additional details on the processing methods and filtering thresholds used. Supplementary Table 2 provides detailed information on selected GEO datasets.

### Cell culture and treatment

The human monocytic cell line THP-1 and the human immortalized B cell line IM-9 were obtained from American Type Culture Collection (USA) and were maintained in RPMI-1640 media (Gibco, USA). The media were supplemented with 1% penicillin-streptomycin and 10% fetal bovine serum (FBS, Gibco, USA), and the cells were incubated in a humidified atmosphere containing 5% CO_2_ at 37° C. To simulate cellular senescence induced by reactive oxygen species (ROS), THP-1 and IM-9 cells were treated with 100 μM and 60 μM H_2_O_2_, respectively, for a period of 24 hours [28, 29].

### Gene expression difference detection and protein-protein interaction (PPI) network construction

We utilized the “limma” package in R to identify senescence-related genes that exhibited significant expression differences between control and sepsis samples. Specifically, we filtered for genes with |log fold change (FC)| > 1 and a false discovery rate (FDR) of < 0.05. Afterwards, we uploaded these differentially-expressed genes to the STRING database (https://cn.string-db.org), with a confidence level set to 0.4, in order to construct a PPI network.

### Feature selection and predictive model construction

We utilized the “glmnet” package to conduct LASSO regression and identify differentially-expressed genes significantly associated with the mortality of sepsis patients. Additionally, we adopted the “randomForestSRC” package to screen for hub genes associated with mortality using the random forest algorithm. To conduct the univariate Cox regression, we utilized the “survival” package, and considered P < 0.01 as significant. The genes co-determined by LASSO, random forest, and univariate Cox regression were then included in multivariate Cox regression analyses with stepwise selection to construct a prognostic model. We defined the risk score calculated by this multivariate Cox regression model as senescence-related score (SRS). To compute SRS, we used the following formula: $\text{SRS}=\sum_{i=1}^{n}Coefif_{i}*Gene_{i}$ where “coeff” represents the coefficient of the gene in the multivariate Cox regression model. More details and parameters regarding these algorithms can be found in Supplementary Material 1.

### Unsupervised clustering

Consensus clustering was performed using the “ConsensusClusterPlus” package in R. The optimal cluster number was determined by analyzing the cumulative distribution function (CDF) curve. Next, principal component analysis (PCA) was carried out to confirm the reliability of the clusters using R’s “prcomp” function.

### Functional annotation and gene set enrichment analysis (GSEA)

The genes were functionally annotated using the Metascape database (https://metascape.org/gp/index.html#/main/step1). GSEA was conducted with version 4.3.2 of the GSEA software, which was downloaded from its official website (https://www.gsea-msigdb.org/gsea/index.jsp). The Hallmark and Reactome gene sets, acquired from the Molecular Signatures Database (https://www.gsea-msigdb.org/gsea/msigdb/index.jsp), served as reference. Significance was determined by terms with nominal P < 0.05 and FDR < 0.25.

### Meta-analyses

To enhance the clarity of diagnosis and prognosis values, we utilized the meta package in R to combine odds ratios (ORs) and hazard ratios (HRs) obtained from several studies. The data from each study were extracted and pooled using either fixed-effect or random-effect models depending on the degree of heterogeneity determined by I-squared (I^2) statistics.

### Kaplan-Meier survival analyses

Kaplan-Meier survival analyses with log-rank tests were performed using the survival package in R. The cut-off value used to divide the GSE65682, GSE95233, and GSE4607 cohorts, which were employed to clarify the prognosis value of SRS in this study into low- and high-SRS subgroups was determined by the median SRS in the training cohort (GSE65682).

### Real-time quantitative PCR (RT-qPCR)

The TRIzol reagent (Invitrogen, USA) was utilized to perform total RNA isolation of the cell samples in accordance with the manufacturer’s protocol. Subsequently, cDNA synthesis was conducted using the PrimeScript RT Reagent Kit (Takara, China), and RT-qPCR experiments were carried out through the use of the Fast SYBR Green Master Mix kit (Takara, China) on the Lightcycler 480 II system (Roche, USA). GAPDH was selected as the internal reference gene to normalize gene expressions. The primer sequence can be found in Supplementary Table 3.

### Statistical analyses

The statistical analyses for the entire study were performed using R software (version 4.2.0), which can be obtained from the official website (https://cran.r-project.org/). The R code used in this study can be found in Supplementary Material 1. Unless otherwise stated, Wilcoxon signed-rank tests were used to compare continuous variables, while student’s t-tests were employed for data obtained from RT-qPCR experiments. Fisher’s exact tests and Pearson Chi-square tests were conducted to evaluate differences in categorical variables across different groups. The pROC package was utilized for receiver operating characteristic (ROC) analyses and calculation of areas under curve (AUCs). A P-value of <0.05 was considered significant in this study. The significance levels were denoted as *P < 0.05; **P < 0.01; ***P < 0.001.

### Availability of data and materials

The raw data can be downloaded from the Gene Expression Omnibus (https://ncbi.nlm.nih.gov/geo/), and the R code used in this study is displayed in Supplementary Material 1.

## RESULTS

### The senescence-related genes exhibiting expression difference

**Figure 1:**
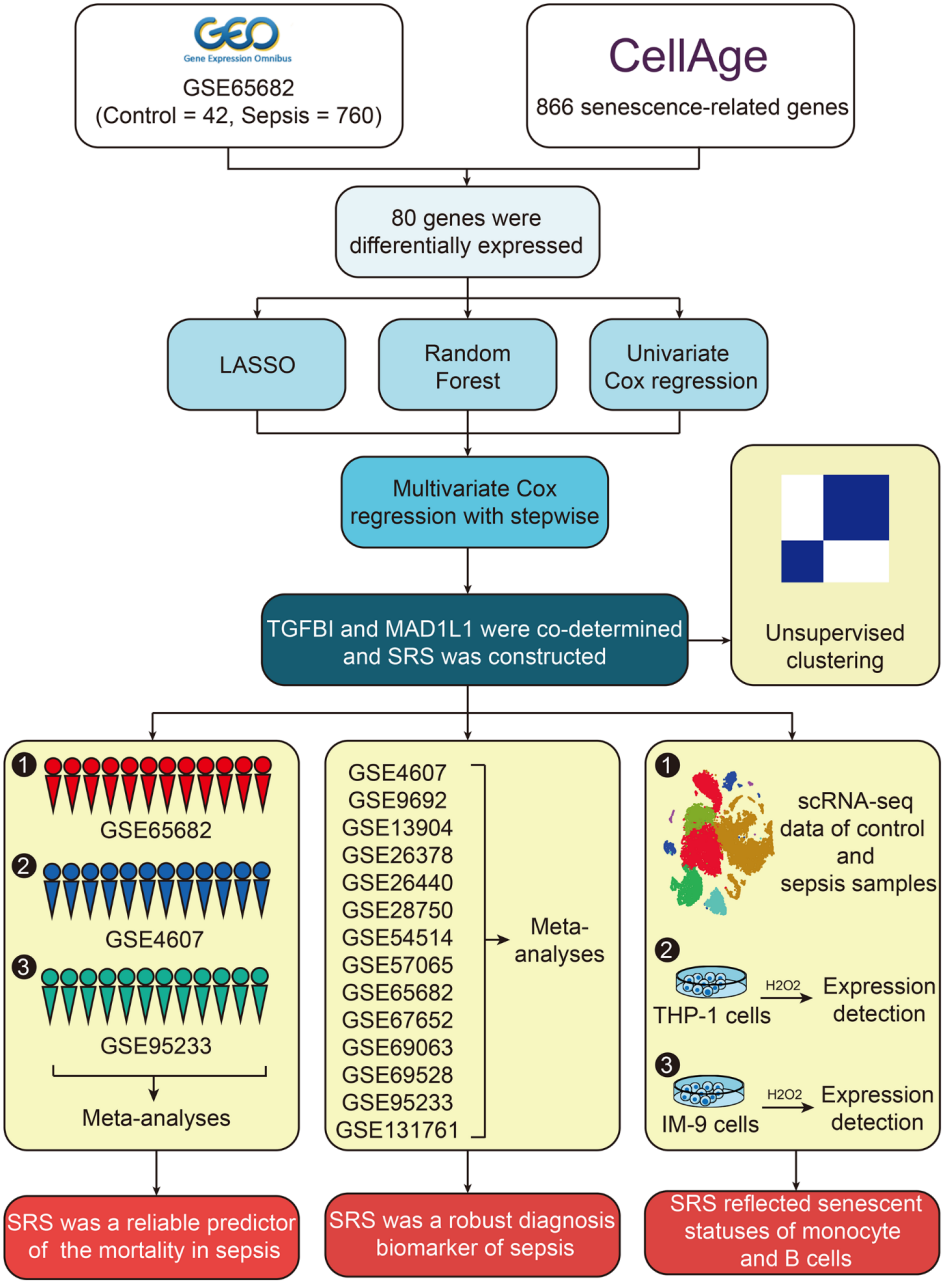
The workflow of this study.

**Figure 2:**
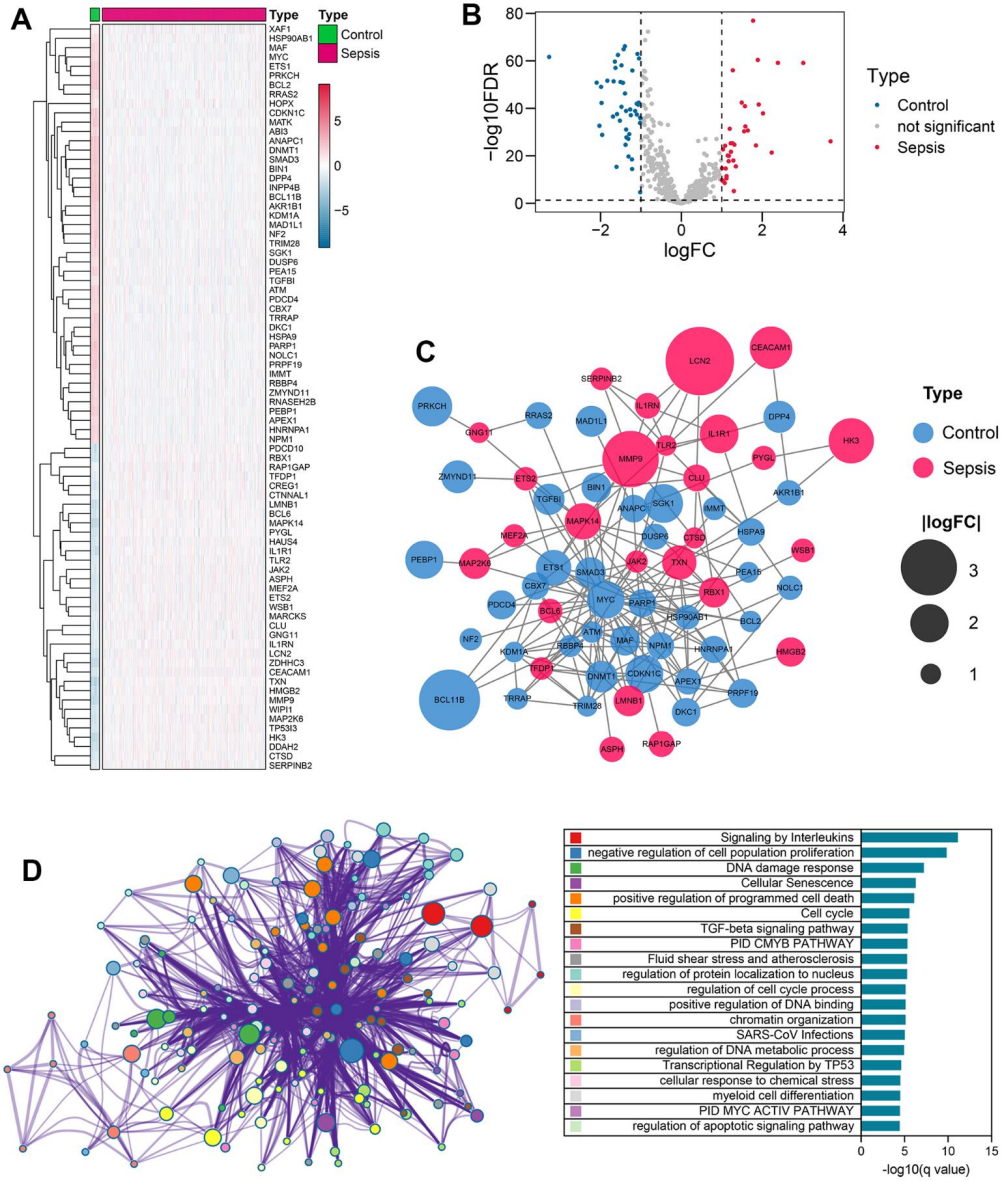
**Differential expression of senescence-related genes in sepsis vs control samples.** (**A**) Heatmap showing the level of expression of 80 senescence-related genes showing expression difference in sepsis and control samples. (**B**) Volcano plot displaying the fold change (log2) and statistical significance (-log10 adjusted p-value) for each gene. The red dots indicate up-regulated genes, while the blue dots indicate down-regulated genes. (**C**) A PPI network of the 80 differentially expressed genes associated with senescence. (**D**) Functional annotation of the 80 differentially expressed genes. Abbreviations: PPI, protein-protein interaction.

The workflow of the present study is illustrated in Figure 1. Firstly, 866 senescence-related genes were collected from CellAge and the GSE65682 dataset was chosen as the training cohort. A total of 80 senescence-related genes showed differential expression between control and sepsis samples (Supplementary Table 4). The heatmap (Figure 2A) and volcano plot (Figure 2B) display the expression levels and the corresponding the fold change (log2) and statistical significance (-log10 FDR) of 80 senescence-related genes, respectively. Subsequently, a protein-protein interaction network was constructed to reveal the underlying interactions among these genes (Figure 2C). Functional annotation demonstrated that these genes were primarily associated with cellular senescence, cell proliferation, cell cycle regulation, DNA damage response and cell apoptosis. Furthermore, some immune-related pathways such as TGF-beta signaling and interleukins signaling pathways were also enriched (Figure 2D), suggesting the potential functions of these genes in sepsis pathogenesis.

### TGFBI and MAD1L1 were significantly associated with the mortality of sepsis

**Figure 3:**
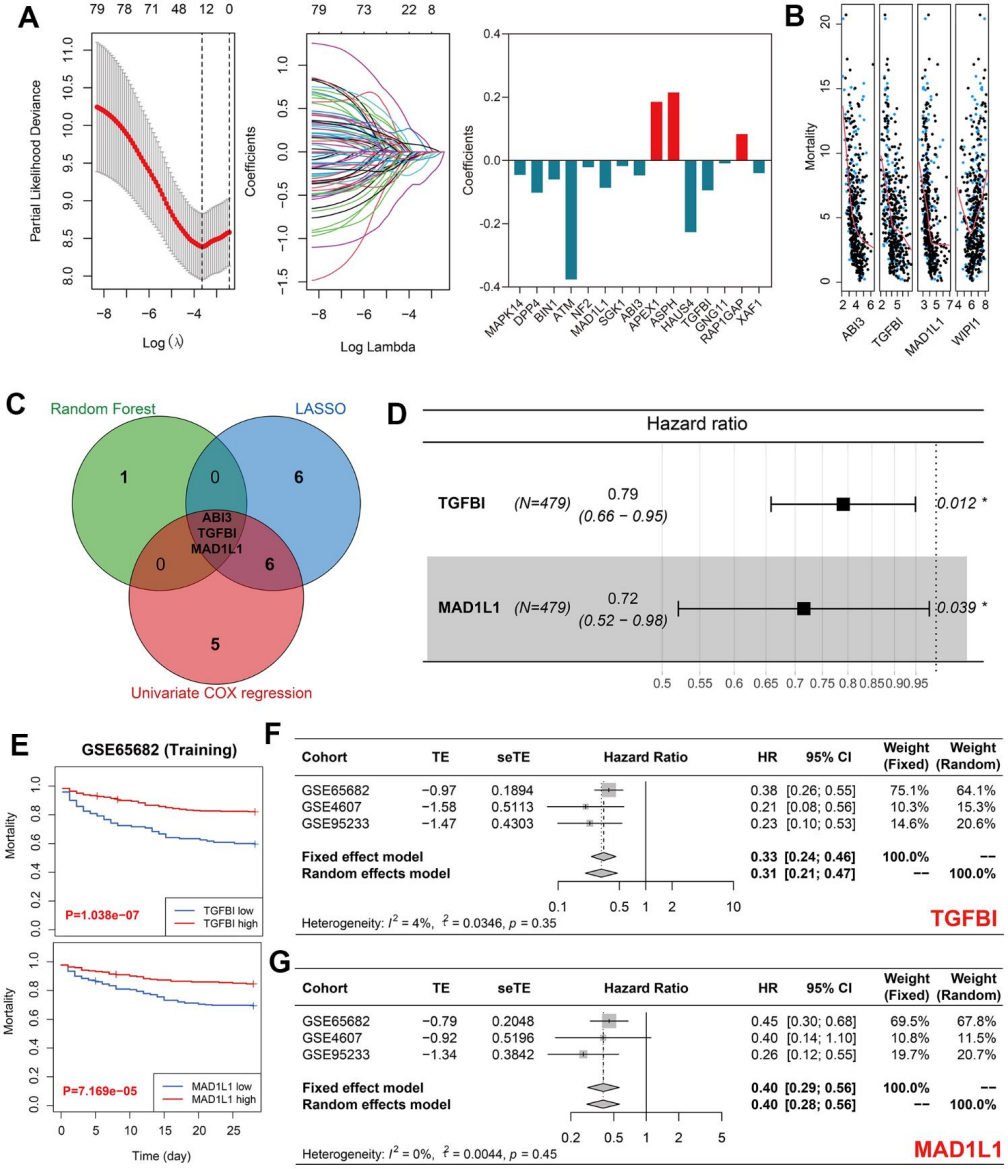
**Identification of TGFBI and MAD1L1 as significant predictors of sepsis mortality.** (**A**) LASSO regression identified 15 out of 80 genes as significant predictors of sepsis mortality. (**B**) Random forest analysis identified four genes, including ABI3, TGFBI, MAD1L1, and WIPI1, as significant predictors of sepsis mortality. (**C**) LASSO, random forest, and univariate Cox analyses identified ABI3, TGFBI, and MAD1L1 as co-determined predictors of sepsis mortality. (**D**) Multivariate Cox regression with stepwise selection ultimately included TGFBI and MAD1L1 in the predictive model for sepsis mortality. (**E**) High expression levels of TGFBI (up) and low expression levels of MAD1L1 (down) were associated with favorable prognoses in the training cohort. (**F**, **G**) Meta-analyses indicated the prognostic value of TGFBI (**F**) and MAD1L1 (**G**) in predicting sepsis mortality. Abbreviations: LASSO, Least Absolute Shrinkage and Selection Operator; TGFBI, transforming growth factor-beta induced protein; MAD1L1, mitotic spindle assembly checkpoint protein.

LASSO regression identified 15 out of 80 differentially expressed genes as significant predictors for sepsis mortality (Figure 3A and Supplementary Table 5). In addition, four genes, namely ABI3, TGFBI, MAD1L1, and WIPI1, were identified by random forest analyses (Figure 3B). 14 out of the total 80 genes were selected through univariate Cox regression with P < 0.01 filtering (Supplementary Table 6). Ultimately, ABI3, TGFBI, and MAD1L1 were co-determined by LASSO analysis, random forest analysis, and univariate Cox analysis (Figure 3C). By using multivariate Cox regression with stepwise function, TGFBI and MAD1L1 were included in the predictive model (Figure 3D), from which SRS was calculated as follows: SRS = -0.791*expression (TGFBI) – 1.036*expression (MAD1L1). The high expression levels of TGFBI and MAD1L1 indicated favorable prognoses in the training cohort (both P < 0.001, Figure 3E). This conclusion was then validated through meta-analyses across the training cohort as well as GSE4607 and GSE95233 cohorts (Figure 3F, 3G; TGFBI, HR = 0.33, 95% confidence interval [CI] = 0.24-0.46; MAD1L1, HR = 0.40, 95% CI = 0.28-0.56).

### Unsupervised clustering based on the expressions of TGFBI and MAD1L1

**Figure 4:**
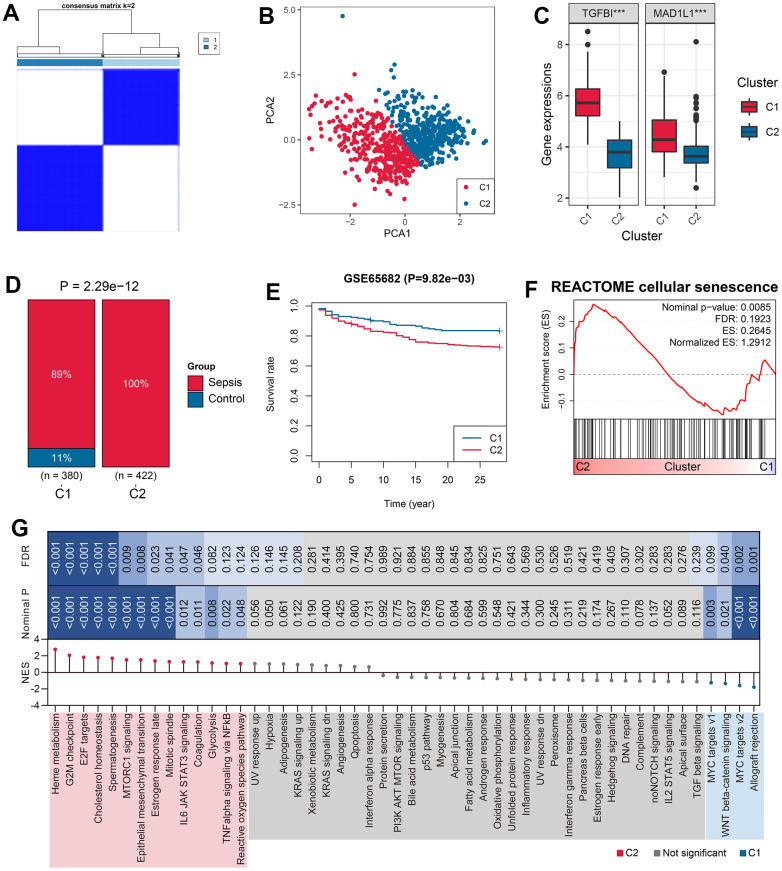
**Unsupervised clustering based on TGFBI and MAD1L1 expression.** (**A**) The consensus clustering algorithm divided 802 samples in the training cohort into two subgroups, C1 and C2. (**B**) PCA was performed to validate the robustness of the clustering. (**C**) The expression levels of TGFBI (left) and MAD1L1 (right) were compared between C1 and C2 subgroups. (**D**) The clustering was associated with sepsis characteristics. (**E**) Subjects in the C2 subgroup exhibited worse prognoses than those in C1 subgroup. (**F**) The clustering was associated with cellular senescence levels. (**G**) Signaling pathways enriched in C1 and C2 samples were identified. Abbreviations: PCA, principal component analysis; C1, cluster 1; C2, cluster 2; ***P < 0.001.

The 802 samples in the GSE65682 cohort were divided into two clusters, Cluster 1 (C1) and Cluster 2 (C2), according to the TGFBI and MAD1L1-based consensus clustering method (Figure 4A and Supplementary Table 7). The clustering results were confirmed by PCA (Figure 4B). The expression levels of TGFBI (P < 0.001) and MAD1L1 (P < 0.001) were significantly downregulated in the C1 subgroup (Figure 4C). Additionally, cases in the C2 subgroup exhibited more sepsis characteristics (P < 0.001, Figure 4D), worse clinical outcomes (P < 0.01, Figure 4E), and a higher level of cellular senescence response (Nominal P < 0.01, FDR < 0.25, Figure 4F). Similar conclusions can be drawn from the analysis of GSE4607 (Supplementary Figure 1) and GSE95233 cohorts (Supplementary Figure 2).

To investigate the differences in pathological mechanisms between C1 and C2 subjects, GSEA was conducted using Hallmark gene sets as reference. Some signaling pathways such as reactive oxygen species (ROS) pathway, IL6 JAK STAT3 signaling, and TNF-alpha signaling via NFkB were enriched in C2 subjects, partly accounting for the unfavorable prognosis for these cases (Figure 4G).

### SRS was a reliable predictor for the mortality of sepsis

**Figure 5:**
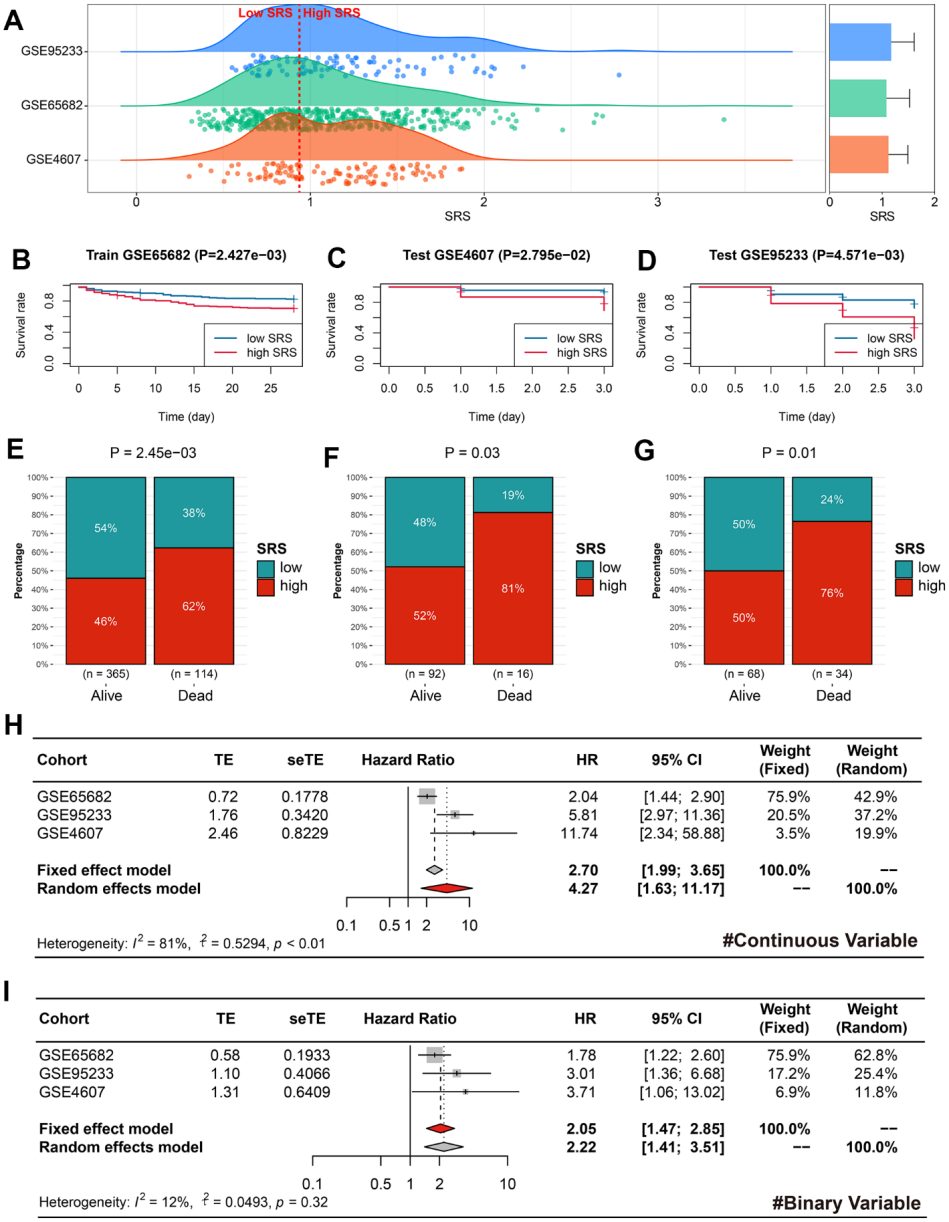
**SRS was a reliable predictor of prognosis in sepsis.** (**A**) Cases in the training, GSE95233, and GSE4607 cohorts were divided into high- and low-SRS subgroups based on the same cut-off value. (**B**–**D**) Kaplan-Meier survival analyses indicated the prognostic value of SRS for sepsis in the training (**B**), GSE4607 (**C**), and GSE95233 (**D**) cohorts. (**E**–**G**) SRS was associated with survival status in the training (**E**), GSE4607 (**F**), and GSE95233 (**G**) cohorts. (**H**, **I**) Meta-analyses were conducted to combine effect values using continuous SRS (**H**) and binary SRS (**I**). Abbreviations: SRS, senescence-related score.

The sepsis subjects in the training, GSE95233, and GSE4607 cohorts were classified into the low- and high-SRS subgroups according to the median SRS level (0.99) in the training cohort (Figure 5A). The high level of SRS exhibited unfavorable clinical outcomes in the training (P < 0.01, Figure 5B), GSE4607 (P < 0.05, Figure 5C), and GSE95233 (P < 0.01, Figure 5D) cohorts. Additionally, more deaths can be observed in the high-SRS subjects from the training (P < 0.01, Figure 5E), GSE4607 (P < 0.05, Figure 5F), and GSE95233 (P < 0.05, Figure 5G) cohorts. Finally, meta-analyses were performed to clarify the predictive ability of SRS on mortality using both continuous and binary SRS measures. The results showed that SRS was a significant prognosis predictor, with a pooled HR of 4.27 (95% CI = 1.63-11.17) for continuous SRS (Figure 5H), and a pooled HR of 2.05 (95% CI = 1.47-2.85) for binary SRS (Figure 5I).

**Figure 6:**
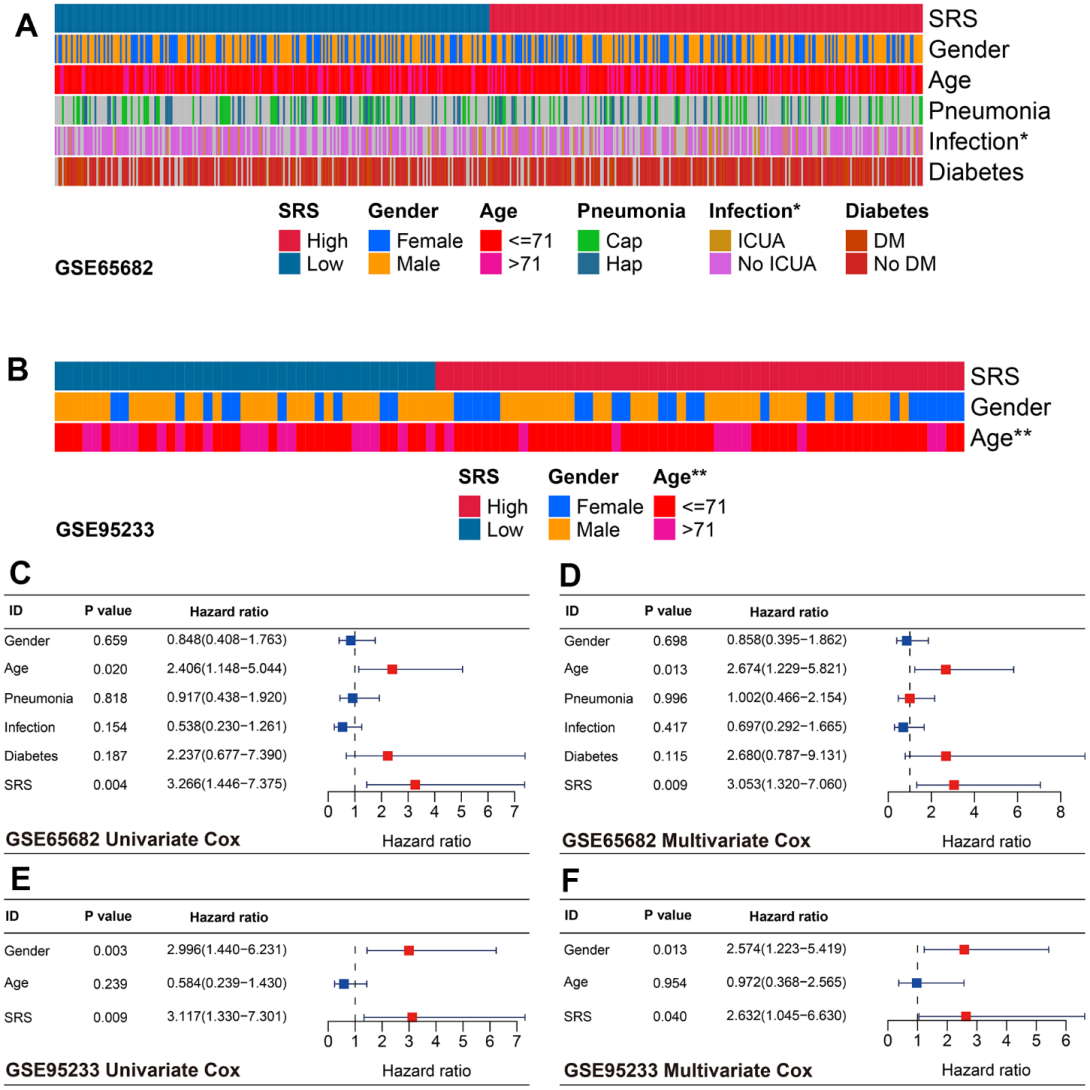
**Association between SRS and clinical features in sepsis.** (**A**) SRS was associated with ICU infection condition in the training cohort. (**B**) SRS was linked with age in the GSE95233 cohort. (**C**, **D**) SRS was an independent predictor of prognosis in both univariate (**C**) and multivariate (**D**) Cox analyses in the training cohort. (**E**, **F**) SRS was an independent predictor of prognosis in both univariate (**E**) and multivariate (**F**) Cox analyses in the GSE95233 cohort. *P < 0.05, **P < 0.01.

The clinical association analyses indicated that SRS was associated with ICU infection status (P < 0.05, Figure 6A) and age (P < 0.01, Figure 6B) in the training and GSE95233 cohorts, respectively. Furthermore, SRS was found to be an independent predictor of mortality through univariate and multivariate Cox analyses in both the training (both P < 0.01, Figure 6C, 6D) and GSE95233 cohorts (both P < 0.05, Figure 6E, 6F), after transforming continuous variables into binary variables.

### SRS was a robust diagnostic biomarker of sepsis

**Figure 7:**
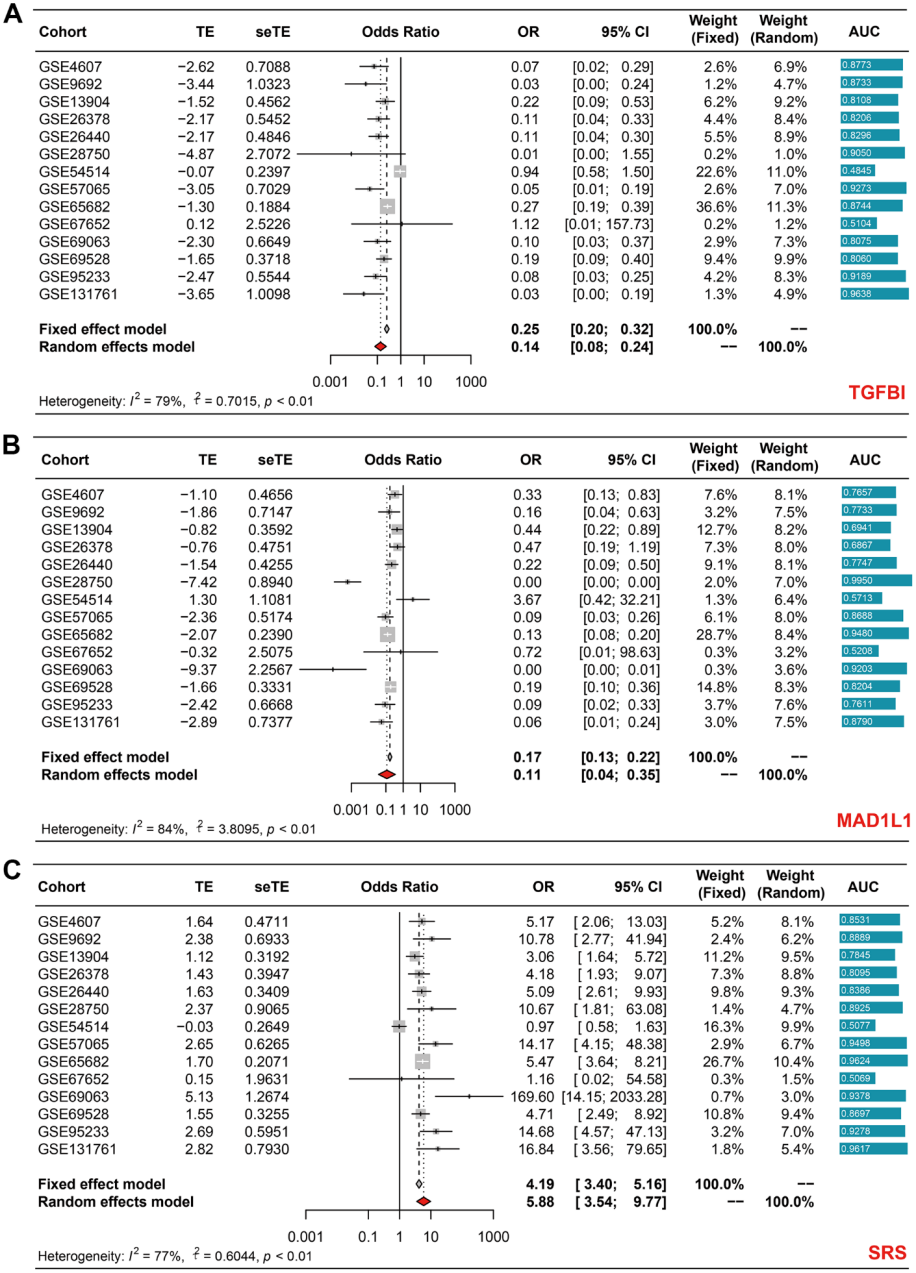
Meta-analyses revealing the diagnostic ability of TGFBI (**A**), MAD1L1 (**B**), and SRS (**C**) in sepsis.

After observing the satisfying performance of SRS in predicting prognosis, we proceeded to investigate whether SRS could also function as a diagnostic biomarker for sepsis using 14 cohorts collected from the GEO database. Our meta-analyses revealed that both TGFBI (pooled OR = 0.14, 95% CI = 0.08-0.24, Figure 7A) and MAD1L1 (pooled OR = 0.11, 95% CI = 0.04-0.35, Figure 7B) had significant diagnostic value. Interestingly, their combination, SRS, showed impressive diagnostic abilities with a pooled OR of 5.88 (95% CI = 3.54-9.77), as displayed in Figure 7C. Supplementary Table 8 presents the AUCs that indicate the diagnostic value of TGFB1, MAD1L1, and SRS in these selected cohorts.

### SRS was associated with the senescence of B cell and monocyte in sepsis

**Figure 8:**
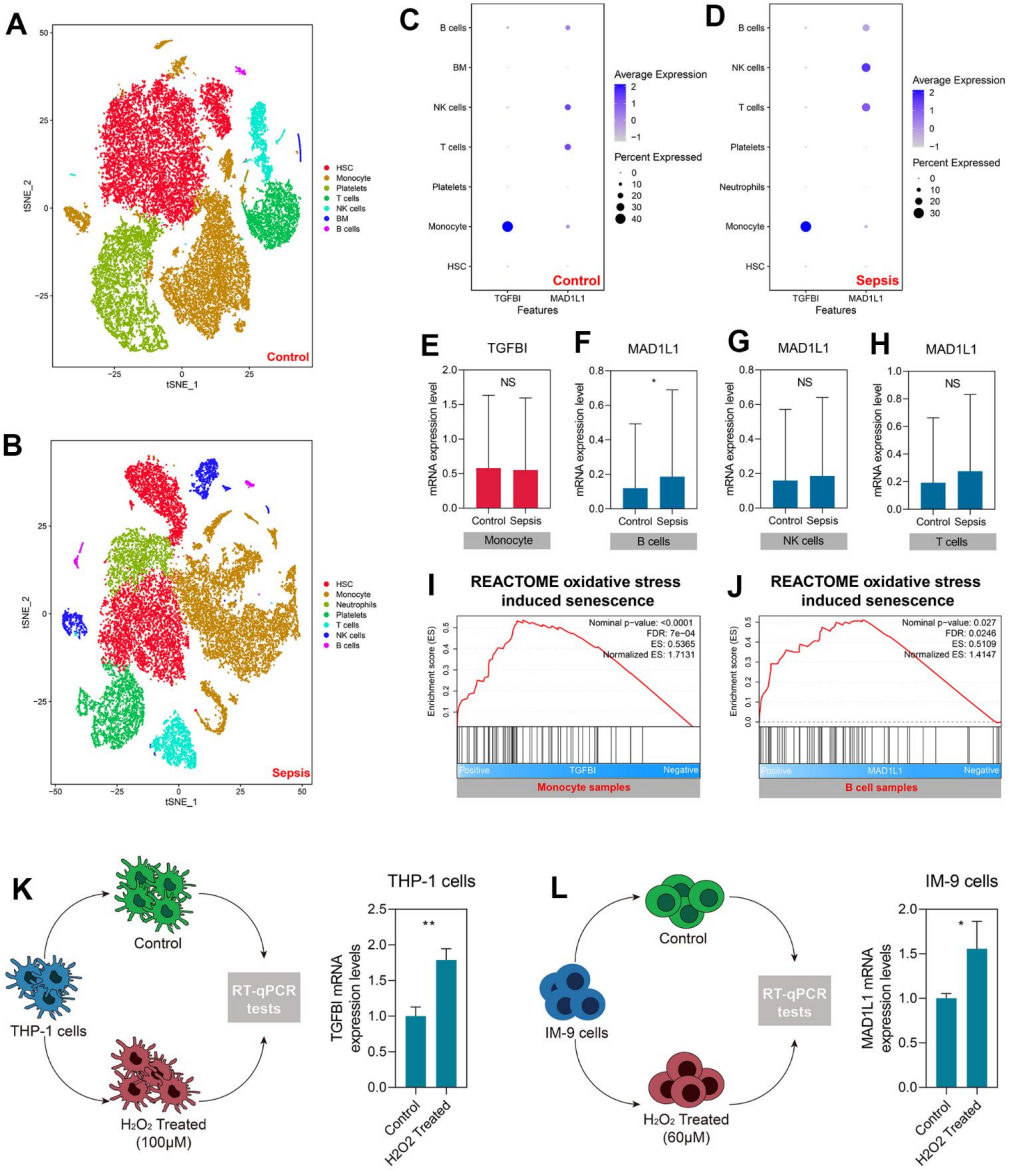
**TGFBI and MAD1L1 were associated with monocyte and B cell senescence, respectively.** (**A**, **B**) Clustering and cell type annotation of cells isolated from healthy control (**A**) and sepsis (**B**) subjects. (**C**, **D**) Levels of TGFBI and MAD1L in different cells isolated from control (**C**) and sepsis (**D**) subjects. (**E**–**H**) Levels of TGFBI in monocytes from control and sepsis subjects (**E**), and levels of MAD1L1 in B cells (**F**), NK cells (**G**), and T cells (**H**) from control and sepsis subjects. (**I**, **J**) Positive association between TGFBI with oxidative stress-induced senescence in monocytes (**I**), and MAD1L with oxidative stress-induced senescence in B cells (**J**). (**K**, **L**) Up-regulation of TGFBI in THP-1 cells treated with H2O2 (**K**), and up-regulation of MAD1L1 in IM-9 cells treated with H2O2 (**L**). Abbreviations: scRNA-seq: single-cell RNA sequencing; *P < 0.05; **P < 0.01; NS: not significant.

We utilized a total of 27,808 cell samples from five healthy control subjects and 21,644 cell samples from four sepsis patients to investigate the mechanisms underlying SRS at a higher resolution. The cell samples obtained from healthy control subjects were segregated into seven main cell types, namely HSC, monocyte, platelets, T cells, NK cells, BM, and B cells (Figure 8A). The cell samples collected from sepsis patients were grouped into HSC, monocyte, neutrophils, platelets, T cells, NK cells, and B cells (Figure 8B). We analyzed the expression levels of TGFBI and MAD1L1 in both the control and sepsis samples (Figure 8C, 8D). Our analysis revealed that TGFBI was predominantly expressed in monocytes; however, there was no significant difference in the expression levels between monocytes obtained from control and sepsis subjects (P > 0.05, Figure 8E). On the other hand, MAD1L1 was found to be highly expressed in B cells as well as NK and T cells. However, only B cells exhibited significant differences in MAD1L1 expression between control and sepsis subjects (P < 0.05, Figure 8F), while no significant differences were observed for MAD1L1 expression between NK cells (P > 0.05, Figure 8G) or T cells (P > 0.05, Figure 8H). Consequently, we investigated the relationship of TFGBI with monocyte senescence while exploring the association of MAD1L1 with B cell senescence.

We assumed that TGFBI and MAD1L1 were linked to oxidative stress-induced cellular senescence in monocytes and B cells, respectively. Subsequently, we conducted GSEA after dividing the monocytes or B cells from sepsis samples into low- and high-expression subgroups based on the median expression level of TGFBI or MAD1L1. Our analysis revealed a positive association between TGFBI and oxidative stress-induced senescence in monocytes (Nominal P < 0.001, FDR < 0.001, Figure 8I). Additionally, we found a similar positive association between MAD1L1 and B cells (Nominal P < 0.05, FDR < 0.05, Figure 8J). We then proceeded to validate these findings experimentally by studying THP-1 and IM-9 cells that were treated with H_2_O_2_. Our observations demonstrated an upregulation of both TGFBI (P < 0.01, Figure 8K) and MAD1L1 (P < 0.05, Figure 8L) expressions in these treated cells.

**Figure 9:**
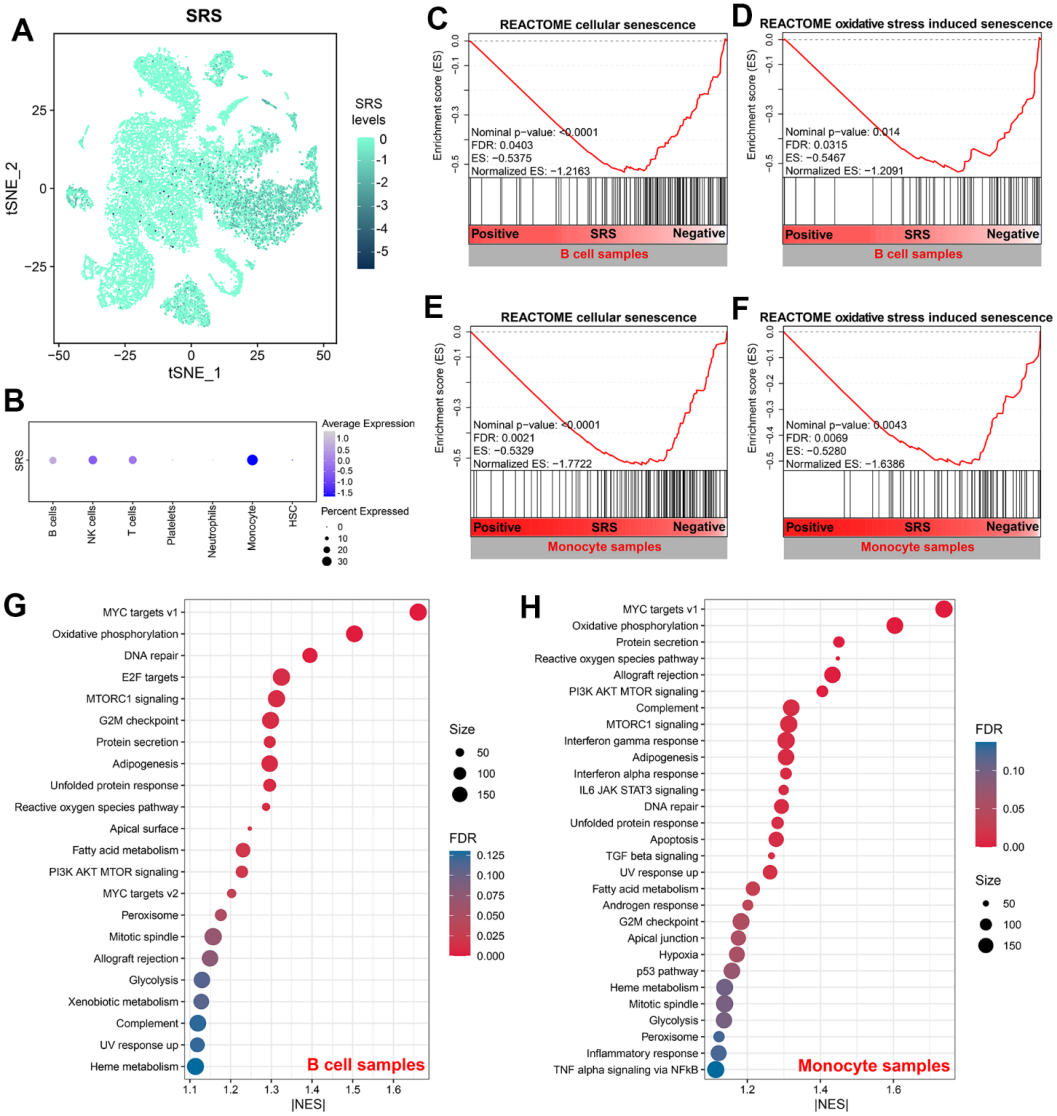
**SRS was associated with cellular senescence in B cells and monocytes.** (**A**) Distribution of SRS levels in cell samples isolated from sepsis subjects. (**B**) Levels of SRS in each cell type. (**C**, **D**) Negative association between SRS and cellular senescence (**C**) and oxidative stress-induced senescence (**D**) in B cells. (**E**, **F**) Negative association between SRS and cellular senescence (**E**) and oxidative stress-induced senescence (**F**) in monocytes. (**G**, **H**) Gene set enrichment analyses showing signaling pathways associated with SRS in B cells (**G**) and monocytes (**H**).

Figure 9A depicts the distribution of SRS in cell samples isolated from sepsis patients. Additionally, Figure 9B displays the levels of SRS in each cell type. SRS was significantly associated with cellular senescence and oxidative stress-induced senescence in B cells (both Nominal P < 0.05, both FDR < 0.05, Figure 9C, 9D) and monocytes (both Nominal P < 0.01, both FDR < 0.01, Figure 9E, 9F). Furthermore, molecular mechanisms were investigated through GSEA in B cells (Figure 9G) and monocytes (Figure 9H). Several pathways such as MYC target V1 were found to be significantly enriched, indicating the underlying biological processes linked with SRS.

## DISCUSSION

Sepsis is a severe medical condition that can occur when the body’s immune system overreacts to an infection, and its prognosis is generally poor. Thus, it is crucial to recognize and treat sepsis early to improve patient outcomes [30]. With the advancement of genomic sequencing technologies, there has been a growing interest in developing novel diagnostic and prognostic gene signatures for sepsis. Although many efforts have been made to improve the efficacy of such signatures [31–33], limited predictive gene signatures have been applied in clinical practice. Cellular senescence has been suggested to contribute to the development of sepsis by releasing pro-inflammatory cytokines and other molecules that can worsen inflammation and organ dysfunction, as discussed above. However, no senescence-related gene signature has been established as a diagnostic or prognostic biomarker for sepsis so far.

The present study established a gene signature related to cellular senescence that included TGFBI and MAD1L1 to evaluate the prognosis and the occurrence in sepsis. This risk score, which we named SRS, demonstrated high prognostic value (pooled HR = 4.27, 95% CI = 1.63-11.17) across three cohorts (GSE65682, GSE95233, and GSE4607). Furthermore, SRS could also serve as a diagnostic tool for sepsis with a pooled OR of 5.88 (95% CI = 3.54-9.77) across 14 sepsis cohorts obtained from the GEO database. Our investigation using scRNA-seq on five healthy controls and four sepsis samples indicated that this gene signature was strongly associated with ROS-induced cellular senescence in both monocytes and B cells. These findings were then validated in THP-1 monocytes and IM-9 B cells *in vitro*.

In our study, we reported for the first time that TGFBI and MAD1L1 could act as diagnostic and prognostic biomarkers in sepsis, and their association with cellular senescence of monocytes and B cells. TGFBI is a gene that encodes an RGD-containing protein located in the extracellular matrix. It plays a critical role in cell proliferation, differentiation, adhesion, migration, and inflammation [34]. A previous study demonstrated that TGFBI could induce cellular senescence in mesothelioma and breast cancer cells [35]. In our study, we observed that TGFBI was associated with ROS-induced cellular senescence processes in monocytes through scRNA-seq analyses and *in vitro* experiments. MAD1L1 encodes a protein serving as a component of the mitotic spindle-assembly checkpoint [36]. In human U-937 myeloid tumor cells, knockdown of MAD1L1 inhibited TGF-beta-induced senescence [37]. Our data showed for the first time that MAD1L1 was linked to ROS-induced cellular senescence in B cells. Our research provides new insights into the mechanisms responsible for sepsis development and may have implications for improving diagnosis and treatment. Our findings show that targeting TGFBI and MAD1L1, given their close association with monocytes and B cells, could be promising therapeutic options for sepsis.

It is important to acknowledge the limitations of this study. Firstly, due to its retrospective nature, the applicability of SRS in clinical practice is limited. To clarify its usefulness, a large-scale, multi-center, double-blind clinical trial is necessary. Secondly, while we demonstrated an association between TGFBI and monocyte senescence as well as MAD1L1 and B cell senescence, further research is needed to elucidate their regulatory relationship and underlying mechanisms. Thirdly, it is crucial to emphasize the significant heterogeneity observed across 14 sepsis cohorts in terms of diagnostic ability. There are several factors that may contribute to this high degree of variation, including differences in the type and severity of the underlying infection, variations in the host’s immune response, and differences in the patient’s genetic makeup and comorbidities. However, due to the unavailability of clinicopathological features in these public datasets, it is impossible to exclude the influence of these factors. Therefore, we were compelled to adopt the random effects model to minimize the impact of these confounding variables as much as possible. In the future, studies should consider stratifying sepsis patients based on relevant factors such as age, comorbidities, severity of infection, and host genetics to create more homogeneous study groups. Additionally, it is important to match control groups with similar characteristics to reduce the impact of confounding variables.

Overall, our study constructed a senescence-related gene signature that can serve as a diagnostic and prognostic predictor for sepsis. This signature offers a potential tool for clinical practice, which could uncover cut-in points to reveal underlying mechanisms and identify novel targets for drug development.

## Supplementary Material

Supplementary Material 1

Supplementary Figures

Supplementary Table 1

Supplementary Tables 2 and 3

Supplementary Table 4

Supplementary Tables 5 and 6

Supplementary Table 7

Supplementary Table 8

## References

[1] Singer M, Deutschman CS, Seymour CW, Shankar-Hari M, Annane D, Bauer M, Bellomo R, Bernard GR, Chiche JD, Coopersmith CM, Hotchkiss RS, Levy MM, Marshall JC, et al. The Third International Consensus Definitions for Sepsis and Septic Shock (Sepsis-3). JAMA. 2016; 315:801–10. 10.1001/jama.2016.0287 26903338 PMC4968574

[2] Weng L, Zeng XY, Yin P, Wang LJ, Wang CY, Jiang W, Zhou MG, Du B, and China Critical Care Clinical Trials Group (CCCCTG). Sepsis-related mortality in China: a descriptive analysis. Intensive Care Med. 2018; 44:1071–80. 10.1007/s00134-018-5203-z 29846748

[3] Rudd KE, Johnson SC, Agesa KM, Shackelford KA, Tsoi D, Kievlan DR, Colombara DV, Ikuta KS, Kissoon N, Finfer S, Fleischmann-Struzek C, Machado FR, Reinhart KK, et al. Global, regional, and national sepsis incidence and mortality, 1990-2017: analysis for the Global Burden of Disease Study. Lancet. 2020; 395:200–11. 10.1016/S0140-6736(19)32989-7 31954465 PMC6970225

[4] Wang J, Sun Y, Teng S, Li K. Prediction of sepsis mortality using metabolite biomarkers in the blood: a meta-analysis of death-related pathways and prospective validation. BMC Med. 2020; 18:83. 10.1186/s12916-020-01546-5 32290837 PMC7157979

[5] Reyes M, Filbin MR, Bhattacharyya RP, Billman K, Eisenhaure T, Hung DT, Levy BD, Baron RM, Blainey PC, Goldberg MB, Hacohen N. An immune-cell signature of bacterial sepsis. Nat Med. 2020; 26:333–40. 10.1038/s41591-020-0752-4 32066974 PMC7235950

[6] Yu R, Wang Y, Liang Q, Xu Y, Yusf AE, Sun L. Identification of potential biomarkers and pathways for sepsis using RNA sequencing technology and bioinformatic analysis. Heliyon. 2023; 9:e15034. 10.1016/j.heliyon.2023.e15034 37089399 PMC10113783

[7] Guan L, Crasta KC, Maier AB. Assessment of cell cycle regulators in human peripheral blood cells as markers of cellular senescence. Ageing Res Rev. 2022; 78:101634. 10.1016/j.arr.2022.101634 35460888

[8] Lucas V, Cavadas C, Aveleira CA. Cellular Senescence: From Mechanisms to Current Biomarkers and Senotherapies. Pharmacol Rev. 2023; 75:675–713. 10.1124/pharmrev.122.000622 36732079

[9] Li X, Li C, Zhang W, Wang Y, Qian P, Huang H. Inflammation and aging: signaling pathways and intervention therapies. Signal Transduct Target Ther. 2023; 8:239. 10.1038/s41392-023-01502-8 37291105 PMC10248351

[10] Martin GS, Mannino DM, Moss M. The effect of age on the development and outcome of adult sepsis. Crit Care Med. 2006; 34:15–21. 10.1097/01.ccm.0000194535.82812.ba 16374151

[11] Quintano Neira RA, Hamacher S, Japiassú AM. Epidemiology of sepsis in Brazil: Incidence, lethality, costs, and other indicators for Brazilian Unified Health System hospitalizations from 2006 to 2015. PLoS One. 2018; 13:e0195873. 10.1371/journal.pone.0195873 29652944 PMC5898754

[12] Chen Q, Mo W. Senescent cell: the ‘factory of viral amplification’. Trends Microbiol. 2023; 31:421–2. 10.1016/j.tim.2023.02.012 36907744

[13] Gioia U, Tavella S, Martínez-Orellana P, Cicio G, Colliva A, Ceccon M, Cabrini M, Henriques AC, Fumagalli V, Paldino A, Presot E, Rajasekharan S, Iacomino N, et al. SARS-CoV-2 infection induces DNA damage, through CHK1 degradation and impaired 53BP1 recruitment, and cellular senescence. Nat Cell Biol. 2023; 25:550–64. 10.1038/s41556-023-01096-x 36894671 PMC10104783

[14] Claushuis TA, van Vught LA, Scicluna BP, Wiewel MA, Klein Klouwenberg PM, Hoogendijk AJ, Ong DS, Cremer OL, Horn J, Franitza M, Toliat MR, Nürnberg P, Zwinderman AH, et al, and Molecular Diagnosis and Risk Stratification of Sepsis Consortium. Thrombocytopenia is associated with a dysregulated host response in critically ill sepsis patients. Blood. 2016; 127:3062–72. 10.1182/blood-2015-11-680744 26956172

[15] Wong HR, Shanley TP, Sakthivel B, Cvijanovich N, Lin R, Allen GL, Thomas NJ, Doctor A, Kalyanaraman M, Tofil NM, Penfil S, Monaco M, Tagavilla MA, et al, and Genomics of Pediatric SIRS/Septic Shock Investigators. Genome-level expression profiles in pediatric septic shock indicate a role for altered zinc homeostasis in poor outcome. Physiol Genomics. 2007; 30:146–55. 10.1152/physiolgenomics.00024.2007 17374846 PMC2770262

[16] Venet F, Schilling J, Cazalis MA, Demaret J, Poujol F, Girardot T, Rouget C, Pachot A, Lepape A, Friggeri A, Rimmelé T, Monneret G, Textoris J. Modulation of LILRB2 protein and mRNA expressions in septic shock patients and after *ex vivo* lipopolysaccharide stimulation. Hum Immunol. 2017; 78:441–50. 10.1016/j.humimm.2017.03.010 28341250

[17] Cvijanovich N, Shanley TP, Lin R, Allen GL, Thomas NJ, Checchia P, Anas N, Freishtat RJ, Monaco M, Odoms K, Sakthivel B, Wong HR, and Genomics of Pediatric SIRS/Septic Shock Investigators. Validating the genomic signature of pediatric septic shock. Physiol Genomics. 2008; 34:127–34. 10.1152/physiolgenomics.00025.2008 18460642 PMC2440641

[18] Wong HR, Cvijanovich N, Allen GL, Lin R, Anas N, Meyer K, Freishtat RJ, Monaco M, Odoms K, Sakthivel B, Shanley TP, and Genomics of Pediatric SIRS/Septic Shock Investigators. Genomic expression profiling across the pediatric systemic inflammatory response syndrome, sepsis, and septic shock spectrum. Crit Care Med. 2009; 37:1558–66. 10.1097/CCM.0b013e31819fcc08 19325468 PMC2747356

[19] Wynn JL, Cvijanovich NZ, Allen GL, Thomas NJ, Freishtat RJ, Anas N, Meyer K, Checchia PA, Lin R, Shanley TP, Bigham MT, Banschbach S, Beckman E, Wong HR. The influence of developmental age on the early transcriptomic response of children with septic shock. Mol Med. 2011; 17:1146–56. 10.2119/molmed.2011.00169 21738952 PMC3321808

[20] Wong HR, Cvijanovich N, Lin R, Allen GL, Thomas NJ, Willson DF, Freishtat RJ, Anas N, Meyer K, Checchia PA, Monaco M, Odom K, Shanley TP. Identification of pediatric septic shock subclasses based on genome-wide expression profiling. BMC Med. 2009; 7:34. 10.1186/1741-7015-7-34 19624809 PMC2720987

[21] Sutherland A, Thomas M, Brandon RA, Brandon RB, Lipman J, Tang B, McLean A, Pascoe R, Price G, Nguyen T, Stone G, Venter D. Development and validation of a novel molecular biomarker diagnostic test for the early detection of sepsis. Crit Care. 2011; 15:R149. 10.1186/cc10274 21682927 PMC3219023

[22] Parnell GP, Tang BM, Nalos M, Armstrong NJ, Huang SJ, Booth DR, McLean AS. Identifying key regulatory genes in the whole blood of septic patients to monitor underlying immune dysfunctions. Shock. 2013; 40:166–74. 10.1097/SHK.0b013e31829ee604 23807251

[23] Tabone O, Mommert M, Jourdan C, Cerrato E, Legrand M, Lepape A, Allaouchiche B, Rimmelé T, Pachot A, Monneret G, Venet F, Mallet F, Textoris J. Endogenous Retroviruses Transcriptional Modulation After Severe Infection, Trauma and Burn. Front Immunol. 2019; 9:3091. 10.3389/fimmu.2018.03091 30671061 PMC6331457

[24] Vieira da Silva Pellegrina D, Severino P, Vieira Barbeiro H, Maziero Andreghetto F, Tadeu Velasco I, Possolo de Souza H, Machado MC, Reis EM, Pinheiro da Silva F. Septic Shock in Advanced Age: Transcriptome Analysis Reveals Altered Molecular Signatures in Neutrophil Granulocytes. PLoS One. 2015; 10:e0128341. 10.1371/journal.pone.0128341 26047321 PMC4457834

[25] Pankla R, Buddhisa S, Berry M, Blankenship DM, Bancroft GJ, Banchereau J, Lertmemongkolchai G, Chaussabel D. Genomic transcriptional profiling identifies a candidate blood biomarker signature for the diagnosis of septicemic melioidosis. Genome Biol. 2009; 10:R127. 10.1186/gb-2009-10-11-r127 19903332 PMC3091321

[26] Martínez-Paz P, Aragón-Camino M, Gómez-Sánchez E, Lorenzo-López M, Gómez-Pesquera E, Fadrique-Fuentes A, Liu P, Tamayo-Velasco Á, Ortega-Loubon C, Martín-Fernández M, Gonzalo-Benito H, García-Morán E, Heredia-Rodríguez M, Tamayo E. Distinguishing septic shock from non-septic shock in postsurgical patients using gene expression. J Infect. 2021; 83:147–55. 10.1016/j.jinf.2021.05.039 34144116

[27] Darden DB, Dong X, Brusko MA, Kelly L, Fenner B, Rincon JC, Dirain ML, Ungaro R, Nacionales DC, Gauthier M, Kladde M, Brusko TM, Bihorac A, et al. A Novel Single Cell RNA-seq Analysis of Non-Myeloid Circulating Cells in Late Sepsis. Front Immunol. 2021; 12:696536. 10.3389/fimmu.2021.696536 34484194 PMC8415415

[28] Alshabibi MA, Khatlani T, Abomaray FM, AlAskar AS, Kalionis B, Messaoudi SA, Khanabdali R, Alawad AO, Abumaree MH. Human decidua basalis mesenchymal stem/stromal cells protect endothelial cell functions from oxidative stress induced by hydrogen peroxide and monocytes. Stem Cell Res Ther. 2018; 9:275. 10.1186/s13287-018-1021-z 30359307 PMC6202803

[29] Zhou R, Zhou J, Muhuitijiang B, Tan W. Construction and experimental validation of a B cell senescence-related gene signature to evaluate prognosis and immunotherapeutic sensitivity in bladder cancer. Funct Integr Genomics. 2022; 23:3. 10.1007/s10142-022-00936-7 36527532

[30] Adams R, Henry KE, Sridharan A, Soleimani H, Zhan A, Rawat N, Johnson L, Hager DN, Cosgrove SE, Markowski A, Klein EY, Chen ES, Saheed MO, et al. Prospective, multi-site study of patient outcomes after implementation of the TREWS machine learning-based early warning system for sepsis. Nat Med. 2022; 28:1455–60. 10.1038/s41591-022-01894-0 35864252

[31] Lin S, Li P, Yang J, Liu S, Huang S, Huang Z, Zhou C, Liu Y. An immune genes signature for predicting mortality in sepsis patients. Front Immunol. 2023; 14:1000431. 10.3389/fimmu.2023.1000431 36860871 PMC9968838

[32] Song J, Ren K, Zhang D, Lv X, Sun L, Deng Y, Zhu H. A novel signature combing cuproptosis- and ferroptosis-related genes in sepsis-induced cardiomyopathy. Front Genet. 2023; 14:1170737. 10.3389/fgene.2023.1170737 37035738 PMC10076593

[33] Chen Z, Chen R, Ou Y, Lu J, Jiang Q, Liu G, Wang L, Liu Y, Zhou Z, Yang B, Zuo L. Construction of an HLA Classifier for Early Diagnosis, Prognosis, and Recognition of Immunosuppression in Sepsis by Multiple Transcriptome Datasets. Front Physiol. 2022; 13:870657. 10.3389/fphys.2022.870657 35685286 PMC9171028

[34] Janša V, Pušić Novak M, Ban Frangež H, Rižner TL. TGFBI as a candidate biomarker for non-invasive diagnosis of early-stage endometriosis. Hum Reprod. 2023; 38:1284–96. 10.1093/humrep/dead091 37187159 PMC10320490

[35] Li B, Wen G, Zhao Y, Tong J, Hei TK. The role of TGFBI in mesothelioma and breast cancer: association with tumor suppression. BMC Cancer. 2012; 12:239. 10.1186/1471-2407-12-239 22695319 PMC3480943

[36] Liu X, Xie H, Fu Z, Yao Q, Han T, Zhan D, Lin Z, Zhu H. MAD1L1 and TSNARE gene polymorphisms are associated with schizophrenia susceptibility in the Han Chinese population. BMC Med Genomics. 2021; 14:218. 10.1186/s12920-021-01070-2 34481484 PMC8418747

[37] Wu S, Hultquist A, Hydbring P, Cetinkaya C, Oberg F, Larsson LG. TGF-beta enforces senescence in Myc-transformed hematopoietic tumor cells through induction of Mad1 and repression of Myc activity. Exp Cell Res. 2009; 315:3099–111. 10.1016/j.yexcr.2009.09.009 19766114

