# Supplementary material for: Unlocking the potential of senescence-related gene signature as a diagnostic and prognostic biomarker in sepsis: insights from meta-analyses, single-cell RNA sequencing, and in vitro experiments
Source: Aging (Albany NY). 2024 Feb 26;16(4):3989–4013. doi: 10.18632/aging.205574 (PMC10929830; doi:10.18632/aging.205574)
Supplement: Supplementary Material 1 [file aging-16-205574-s001.docx]

Supplementary Material 1. R codes used in the present study.

#Gene expression difference detection

logFoldChange=0.5

adjustP=0.05

conNum=42

treatNum=760

library(limma)

rt=read.table("all_exp_ranked.txt",sep="\t",header=T,check.names=F)

rt=as.matrix(rt)

rownames(rt)=rt[,1]

exp=rt[,2:ncol(rt)]

dimnames=list(rownames(exp),colnames(exp))

rt=matrix(as.numeric(as.matrix(exp)),nrow=nrow(exp),dimnames=dimnames)

rt=avereps(rt)

modType=c(rep("con",conNum),rep("treat",treatNum))

design <- model.matrix(~0+factor(modType))

colnames(design) <- c("con","treat")

fit <- lmFit(rt,design)

cont.matrix<-makeContrasts(treat-con,levels=design)

fit2 <- contrasts.fit(fit, cont.matrix)

fit2 <- eBayes(fit2)

allDiff=topTable(fit2,adjust='fdr',number=200000)

write.table(allDiff,file="All_limma.xls",sep="\t",quote=F)

#LASSO regression

library("glmnet")

library("survival")

rt=read.table("input.txt",header=T,sep="\t",row.names=1,check.names=F)

rt$futime[rt$futime<=0]=1

x=as.matrix(rt[,c(3:ncol(rt))])

y=data.matrix(Surv(rt$futime,rt$fustat))

fit <- glmnet(x, y, family = "cox", maxit = 1000)

pdf("lambda.pdf",4.5,4.5)

plot(fit, xvar = "lambda", label = TRUE)

dev.off()

cvfit <- cv.glmnet(x, y, family="cox", maxit = 1000)

pdf("cvfit.pdf",4.5,4.5)

plot(cvfit)

abline(v=log(c(cvfit$lambda.min,cvfit$lambda.1se)),lty="dashed")

dev.off()

coef <- coef(fit, s = cvfit$lambda.min)

index <- which(coef != 0)

actCoef <- coef[index]

write.csv(actCoef,"actCoef.csv")

lassoGene=row.names(coef)[index]

lassoGene=c("futime","fustat",lassoGene)

lassoSigExp=rt[,lassoGene]

lassoSigExp=cbind(id=row.names(lassoSigExp),lassoSigExp)

write.table(lassoSigExp,file="lassoSigExp.txt",sep="\t",row.names=F,quote=F)

#random forest analysis

data <- read.table("input.txt",sep="\t",header=T,check.names=F,row.names = 1)

library(randomForestSRC)

library(survival)

rf <- rfsrc(Surv(futime,fustat) ~ .,data = data,ntree = 1000,mtry = NULL, nodesize = NULL,splitrule = "logrank",importance = TRUE)

plot(rf)

result_vimp <- vimp(rf)$importance

write.table(result_vimp, "result_vimp.xls", sep="\t",quote=F,col.names=T)

our.rf <- var.select(object = rf, vdv,

method = "vh.vimp", nrep = 50)

our.rf$rfsrc.refit.obj

topvars <- our.rf$topvars

RF_topvars <- c("futime","fustat")

RF_topvars_exp <- data[,RF_topvars]

pdf("topvars.pdf", 6,5)

plot.variable(our.rf$rfsrc.refit.obj,

xvar.names = our.rf$topvars[1:8], cex.main = 2)

dev.off()

#Univariate Cox regression

pFilter=0.01

library(survival)

rt=read.table("input.txt",header=T,sep="\t",check.names=F,row.names=1)

sigGenes=c("futime","fustat")

outTab=data.frame()

for(i in colnames(rt[,3:ncol(rt)])){

cox <- coxph(Surv(futime, fustat) ~ rt[,i], data = rt)

coxSummary = summary(cox)

coxP=coxSummary$coefficients[,"Pr(>|z|)"]

outTab=rbind(outTab,

cbind(id=i,

HR=coxSummary$conf.int[,"exp(coef)"],

HR.95L=coxSummary$conf.int[,"lower .95"],

HR.95H=coxSummary$conf.int[,"upper .95"],

pvalue=coxSummary$coefficients[,"Pr(>|z|)"])

)

if(coxP<pFilter){

sigGenes=c(sigGenes,i)

}

}

write.table(outTab,file="unicox.xls",sep="\t",row.names=F,quote=F)

#Multivariate Cox regression with stepwise and SRS calculation

library(survival)

library(survminer)

rt=read.table("input.txt",header=T,sep="\t",check.names=F,row.names=1)

multiCox=coxph(Surv(futime, fustat) ~ ., data = rt)

multiCox=step(multiCox,direction = "both")

multiCoxSum=summary(multiCox)

outTab=data.frame()

outTab=cbind(

coef=multiCoxSum$coefficients[,"coef"],

HR=multiCoxSum$conf.int[,"exp(coef)"],

HR.95L=multiCoxSum$conf.int[,"lower .95"],

HR.95H=multiCoxSum$conf.int[,"upper .95"],

pvalue=multiCoxSum$coefficients[,"Pr(>|z|)"])

outTab=cbind(id=row.names(outTab),outTab)

write.table(outTab,file="multiCox.xls",sep="\t",row.names=F,quote=F)

riskScore=predict(multiCox,type="risk",newdata=rt)

coxGene=rownames(multiCoxSum$coefficients)

coxGene=gsub("`","",coxGene)

outCol=c("futime","fustat",coxGene)

medianTrainRisk=median(riskScore)

risk=as.vector(ifelse(riskScore>medianTrainRisk,"high","low"))

write.table(cbind(id=rownames(cbind(rt[,outCol],riskScore,risk)),cbind(rt[,outCol],riskScore,risk)),

file="riskTrain.txt",

sep="\t",

quote=F,

row.names=F)

rtTest=read.table("test_GSE4607.txt",header=T,sep="\t",check.names=F,row.names=1)

rtTest[,"futime"]=rtTest[,"futime"]/365

riskScoreTest=predict(multiCox,type="risk",newdata=rtTest)

riskTest=as.vector(ifelse(riskScoreTest>medianTrainRisk,"high","low"))

write.table(cbind(id=rownames(cbind(rtTest[,outCol],riskScoreTest,riskTest)),cbind(rtTest[,outCol],riskScore=riskScoreTest,risk=riskTest)),

file="riskTest_GSE4607.txt",

sep="\t",

quote=F,

row.names=F)

rtTest=read.table("test_GSE95233.txt",header=T,sep="\t",check.names=F,row.names=1)

rtTest[,"futime"]=rtTest[,"futime"]/365

riskScoreTest=predict(multiCox,type="risk",newdata=rtTest)

riskTest=as.vector(ifelse(riskScoreTest>medianTrainRisk,"high","low"))

write.table(cbind(id=rownames(cbind(rtTest[,outCol],riskScoreTest,riskTest)),cbind(rtTest[,outCol],riskScore=riskScoreTest,risk=riskTest)),

file="riskTest_GSE95233.txt",

sep="\t",

quote=F,

row.names=F)

#Meta-analysis

data_a <- read.table("INPUT_SRS.txt",sep="\t",header = T, check.names = F,row.names = 1)

head(data_a)

library("meta")

lnor<- log(data_a[,"OR"])

lnuci<- log(data_a[,"OR.95H"])

lnlci<- log(data_a[,"OR.95L"])

selnor<- (lnuci-lnlci)/(2*1.96)

pfs=metagen(lnor,selnor,sm="OR",data=data_a,studlab=paste(data_a$Cohort,sep="-"))

pfs

forest(pfs)

pdf("INPUT_SRS.PDF",12,7)

forest(pfs)

dev.off()

#Kaplan-Meier survival analysis

library(survival)

rt=read.table("riskTrain.txt",header=T,sep="\t")

diff=survdiff(Surv(futime, fustat) ~risk,data = rt)

pValue=1-pchisq(diff$chisq,df=1)

pValue=signif(pValue,4)

pValue=format(pValue, scientific = TRUE)

fit <- survfit(Surv(futime, fustat) ~ risk, data = rt)

summary(fit)

pdf(file="survival_train.pdf",width=4.5,height=4.5)

plot(fit,

lwd=2,

col=c("#E01B3E","#006A9D"),

xlab="Time (year)",

ylab="Survival rate",

main=paste("GSE65682 (P=", pValue ,")",sep=""),

mark.time=T)

legend("topright",

c("high SRS", "low SRS"),

lwd=2,

col=c("#E01B3E","#006A9D"))

dev.off()

#Consensus clustering

workDir="XXX"

setwd(workDir)

rt=read.table("exp.txt",sep="\t",header=T,check.names=F)

rt=as.matrix(rt)

rownames(rt)=rt[,1]

exp=rt[,2:ncol(rt)]

dimnames=list(rownames(exp),colnames(exp))

data=matrix(as.numeric(as.matrix(exp)),nrow=nrow(exp),dimnames=dimnames)

data=avereps(data)

maxK=9

library(ConsensusClusterPlus)

results = ConsensusClusterPlus(data,

maxK=maxK,

reps=50,

pItem=0.8,

pFeature=1,

title=workDir,

clusterAlg="km",

distance="euclidean",

plot="pdf")

clusterNum=2

cluster=results[[clusterNum]][["consensusClass"]]

write.table(cluster,file="cluster.txt",sep="\t",quote=F,col.names=F)

#Principal Component Analysis

library(limma)

rt=read.table("symbol.txt",sep="\t",header=T,check.names=F)

rt=as.matrix(rt)

rownames(rt)=rt[,1]

exp=rt[,2:ncol(rt)]

dimnames=list(rownames(exp),colnames(exp))

data=matrix(as.numeric(as.matrix(exp)),nrow=nrow(exp),dimnames=dimnames)

data=avereps(data)

data=t(data)

data.class <- rownames(data)

data.pca <- prcomp(data, scale. = TRUE)

write.table(predict(data.pca),file="newTab.xls",quote=F,sep="\t")

library(ggplot2)

cluster=read.table("cluster.txt",sep="\t",header=F)

group=paste0("cluster",as.vector(cluster[,2]))

pcaPredict=predict(data.pca)

PCA = data.frame(PCA1 = pcaPredict[,1], PCA2 = pcaPredict[,2],group=group)

pdf(file="PCA.pdf",height=5,width=6.5)

ggplot(data = PCA, aes(PCA1, PCA2)) + geom_point(aes(color = group)) +

theme_bw()+

theme(plot.margin=unit(rep(1.5,4),'lines'))+

theme(panel.grid.major = element_blank(), panel.grid.minor = element_blank())

dev.off()

#Chi-Square test

library(ggstatsplot)

library(ggplot2)

data <- read.table("input.txt",sep = "\t",header = T,row.names = 1)

A <- table(data)

chisq.test(A)

pdf("input.pdf",4,4)

ggstatsplot::ggbarstats(data = data,

x = Group,

y = Cluster,

xlab = NULL,

ylab = "Percentage")

dev.off()

#Wilcoxon signed-rank test

library(ggplot2)

library(reshape2)

inputFile="input.txt"

outFile="boxplot.pdf"

rt=read.table(inputFile, header=T,sep="\t",check.names=F,row.names=1)

x=colnames(rt)[1]

colnames(rt)[1]="Type"

geneSig=c("")

for(gene in colnames(rt)[2:ncol(rt)]){

rt1=rt[,c(gene,"Type")]

colnames(rt1)=c("expression","Type")

p=1

if(length(levels(factor(rt1$Type)))>2){

test=kruskal.test(expression ~ Type, data = rt1)

p=test$p.value

}else{

test=wilcox.test(expression ~ Type, data = rt1)

p=test$p.value

}

Sig=ifelse(p<0.001,"***",ifelse(p<0.01,"**",ifelse(p<0.05,"*","")))

geneSig=c(geneSig,Sig)

}

colnames(rt)=paste0(colnames(rt),geneSig)

data=melt(rt,id.vars=c("Type"))

colnames(data)=c("Type","Gene","Expression")

p1=ggplot(data,aes(x=Type,y=Expression,fill=Type))+

guides(fill=guide_legend(title=x))+

labs(x = x, y = "Gene expressions")+

geom_boxplot()+ facet_wrap(~Gene,nrow =1)+ theme_bw()+

theme(axis.text.x = element_text(angle = 45, hjust = 1))

pdf(file=outFile, width=9, height=3)

print(p1)

dev.off()

#Odds ratios calculation and ROC analyses

library(rms)

library(pROC)

rt<-read.table("input.txt",header=T,sep="\t",check.names=F,row.names=1)

ddist <- datadist(rt)

options(datadist="ddist")

pFilter <- 0.05

sigGenes=c("status")

outTab=data.frame()

for(i in colnames(rt[,2:ncol(rt)])){

mymodel <- glm(status~rt[,i],family=binomial(link = "logit"),data = rt)

mymodelSummary = summary(mymodel)

mymodelP=mymodelSummary$coefficients[2,"Pr(>|z|)"]

predict <- predict.glm(mymodel,type = "response",newdata = rt)

true_value = rt[,1]

modelroc <- roc(true_value,predict)

AUC = modelroc$auc

outTab=rbind(outTab,

cbind(id=i,

OR=matrix(exp(coefficients(mymodel)))[2,1],

OR.95L=exp(confint(mymodel))[2,"2.5 %"],

OR.95H=exp(confint(mymodel))[2,"97.5 %"],

pvalue=mymodelSummary$coefficients[2,"Pr(>|z|)"],

AUC=AUC)

)

if(mymodelP<pFilter){

sigGenes=c(sigGenes,i)

}

}

write.table(outTab,file="unilogit.xls",sep="\t",row.names=F,quote=F)

#single-cell RNA sequencing analysis

library(Seurat)

logFCfilter=1

adjPvalFilter=0.05

ref=celldex::HumanPrimaryCellAtlasData()

library(limma)

library(Seurat)

library(dplyr)

library(magrittr)

library(celldex)

library(SingleR)

#HC1

dir="D:\\sepsis\\raw_data\\GSE175453_scRNAseq\\HC1"

list.files(dir)

counts <- Read10X(data.dir = dir)

HC1=CreateSeuratObject(counts = counts$"Gene Expression",project = "seurat", min.cells=3, min.features=50, names.delim = "-")

#HC2

dir="D:\\sepsis\\raw_data\\GSE175453_scRNAseq\\HC2"

list.files(dir)

counts <- Read10X(data.dir = dir)

HC2=CreateSeuratObject(counts = counts$"Gene Expression",project = "seurat", min.cells=3, min.features=50, names.delim = "-")

#HC3

dir="D:\\sepsis\\raw_data\\GSE175453_scRNAseq\\HC3"

list.files(dir)

counts <- Read10X(data.dir = dir)

HC3=CreateSeuratObject(counts = counts$"Gene Expression",project = "seurat", min.cells=3, min.features=50, names.delim = "-")

#HC4

dir="D:\\sepsis\\raw_data\\GSE175453_scRNAseq\\HC4"

list.files(dir)

counts <- Read10X(data.dir = dir)

HC4=CreateSeuratObject(counts = counts$"Gene Expression",project = "seurat", min.cells=3, min.features=50, names.delim = "-")

#HC5

dir="D:\\sepsis\\raw_data\\GSE175453_scRNAseq\\HC5"

list.files(dir)

counts <- Read10X(data.dir = dir)

HC5=CreateSeuratObject(counts = counts$"Gene Expression",project = "seurat", min.cells=3, min.features=50, names.delim = "-")

setwd("D:\\sepsis\\raw_data\\GSE175453_scRNAseq\\HC_ALL")

#MERGE

pbmc <- merge(HC1, y = c(HC2, HC3, HC4, HC5), add.cell.ids = c("HC1", "HC2", "HC3", "HC4", "HC5"), project = "pbmc")

str(pbmc)

exp_HC <- GetAssayData(object = pbmc, slot = "counts")

dim(exp_HC)

exp_HC[1:4,1:4]

write.csv(exp_HC, "all_exp_HC.csv")

pbmc[["percent.mt"]] <- PercentageFeatureSet(object = pbmc, pattern = "^MT-")

pdf(file="01.featureViolin.pdf", width=10, height=6)

VlnPlot(object = pbmc, features = c("nFeature_RNA", "nCount_RNA", "percent.mt"), ncol = 3)

dev.off()

pbmc=subset(x = pbmc, subset = nFeature_RNA > 50 & percent.mt < 5)

pdf(file="01.featureCor.pdf",width=10,height=6)

plot1 <- FeatureScatter(object = pbmc, feature1 = "nCount_RNA", feature2 = "percent.mt",pt.size=1.5)

plot2 <- FeatureScatter(object = pbmc, feature1 = "nCount_RNA", feature2 = "nFeature_RNA",pt.ize=1.5)

CombinePlots(plots = list(plot1, plot2))

dev.off()

pbmc <- NormalizeData(object = pbmc, normalization.method = "LogNormalize", scale.factor = 10000)

pbmc <- FindVariableFeatures(object = pbmc, selection.method = "vst", nfeatures = 1500)

top10 <- head(x = VariableFeatures(object = pbmc), 10)

pdf(file="01.featureVar.pdf",width=10,height=6)

plot1 <- VariableFeaturePlot(object = pbmc)

plot2 <- LabelPoints(plot = plot1, points = top10, repel = TRUE)

CombinePlots(plots = list(plot1, plot2))

dev.off()

pbmc=ScaleData(pbmc)

pbmc=RunPCA(object= pbmc,npcs = 20,pc.genes=VariableFeatures(object = pbmc))

pdf(file="02.pcaGene.pdf",width=10,height=8)

VizDimLoadings(object = pbmc, dims = 1:4, reduction = "pca",nfeatures = 20)

dev.off()

pdf(file="02.PCA.pdf",width=6.5,height=6)

DimPlot(object = pbmc, reduction = "pca")

dev.off()

pdf(file="02.pcaHeatmap.pdf",width=10,height=8)

DimHeatmap(object = pbmc, dims = 1:4, cells = 500, balanced = TRUE,nfeatures = 30,ncol=2)

dev.off()

pbmc <- JackStraw(object = pbmc, num.replicate = 100)

pbmc <- ScoreJackStraw(object = pbmc, dims = 1:15)

pdf(file="02.pcaJackStraw.pdf",width=8,height=6)

JackStrawPlot(object = pbmc, dims = 1:15)

dev.off()

pcSelect=14

pbmc <- FindNeighbors(object = pbmc, dims = 1:pcSelect)

pbmc <- FindClusters(object = pbmc, resolution = 0.5)

pbmc <- RunTSNE(object = pbmc, dims = 1:pcSelect,check_duplicates = FALSE)

pdf(file="03.TSNE.pdf",width=6.5,height=6)

TSNEPlot(object = pbmc, pt.size = 2, label = TRUE)

dev.off()

write.table(pbmc$seurat_clusters,file="03.tsneCluster.txt",quote=F,sep="\t",col.names=F)

pbmc.markers <- FindAllMarkers(object = pbmc,

only.pos = FALSE,

min.pct = 0.25,

logfc.threshold = logFCfilter)

sig.markers=pbmc.markers[(abs(as.numeric(as.vector(pbmc.markers$avg_log2FC)))>logFCfilter & as.numeric(as.vector(pbmc.markers$p_val_adj))<adjPvalFilter),]

write.table(sig.markers,file="03.clusterMarkers.txt",sep="\t",row.names=F,quote=F)

top10 <- pbmc.markers %>% group_by(cluster) %>% top_n(n = 10, wt = avg_log2FC)

pdf(file="03.tsneHeatmap.pdf",width=12,height=9)

DoHeatmap(object = pbmc, features = top10$gene) + NoLegend()

dev.off()

pdf(file="03.markerViolin.pdf",width=10,height=6)

VlnPlot(object = pbmc, features = row.names(sig.markers)[1:2])

dev.off()

showGenes=c("TGFBI","MAD1L1")

pdf(file="03.markerScatter.pdf",width=10,height=6)

FeaturePlot(object = pbmc, features = showGenes, cols = c("green", "red"))

dev.off()

pdf(file="03.markerBubble.pdf",width=12,height=6)

cluster10Marker=showGenes

DotPlot(object = pbmc, features = cluster10Marker)

dev.off()

counts<-pbmc@assays$RNA@counts

clusters<-pbmc@meta.data$seurat_clusters

ann=pbmc@meta.data$orig.ident

#ref=get(load("ref_Human_all.RData"))

#ref=celldex::HumanPrimaryCellAtlasData()

singler=SingleR(test=counts, ref =ref,

labels=ref$label.main, clusters = clusters)

clusterAnn=as.data.frame(singler)

clusterAnn=cbind(id=row.names(clusterAnn), clusterAnn)

clusterAnn=clusterAnn[,c("id", "labels")]

write.table(clusterAnn,file="04.clusterAnn.txt",quote=F,sep="\t", row.names=F)

singler2=SingleR(test=counts, ref =ref,

labels=ref$label.main)

cellAnn=as.data.frame(singler2)

cellAnn=cbind(id=row.names(cellAnn), cellAnn)

cellAnn=cellAnn[,c("id", "labels")]

write.table(cellAnn, file="04.cellAnn.txt", quote=F, sep="\t", row.names=F)

newLabels=singler$labels

names(newLabels)=levels(pbmc)

pbmc=RenameIdents(pbmc, newLabels)

pdf(file="04.TSNE.pdf",width=8,height=8)

TSNEPlot(object = pbmc, pt.size = 0.5, label = TRUE)

dev.off()

pdf(file="04.markerScatter.pdf",width=12,height=6)

FeaturePlot(object = pbmc, features = showGenes, cols = c("green", "red"))

dev.off()

pdf(file="04.markerBubble.pdf",width=6,height=6)

cluster10Marker=showGenes

DotPlot(object = pbmc, features = cluster10Marker)

dev.off()

pbmc.markers=FindAllMarkers(object = pbmc,

only.pos = FALSE,

min.pct = 0.25,

logfc.threshold = logFCfilter)

sig.cellMarkers=pbmc.markers[(abs(as.numeric(as.vector(pbmc.markers$avg_log2FC)))>logFCfilter & as.numeric(as.vector(pbmc.markers$p_val_adj))<adjPvalFilter),]

write.table(sig.cellMarkers,file="04.cellMarkers.txt",sep="\t",row.names=F,quote=F)
