# Supplementary material for: Unlocking the potential of senescence-related gene signature as a diagnostic and prognostic biomarker in sepsis: insights from meta-analyses, single-cell RNA sequencing, and in vitro experiments
Source: Aging (Albany NY). 2024 Feb 26;16(4):3989–4013. doi: 10.18632/aging.205574 (PMC10929830; doi:10.18632/aging.205574)
Supplement: Supplementary Figures [file aging-16-205574-s002.pdf]

SUPPLEMENTARY FIGURES

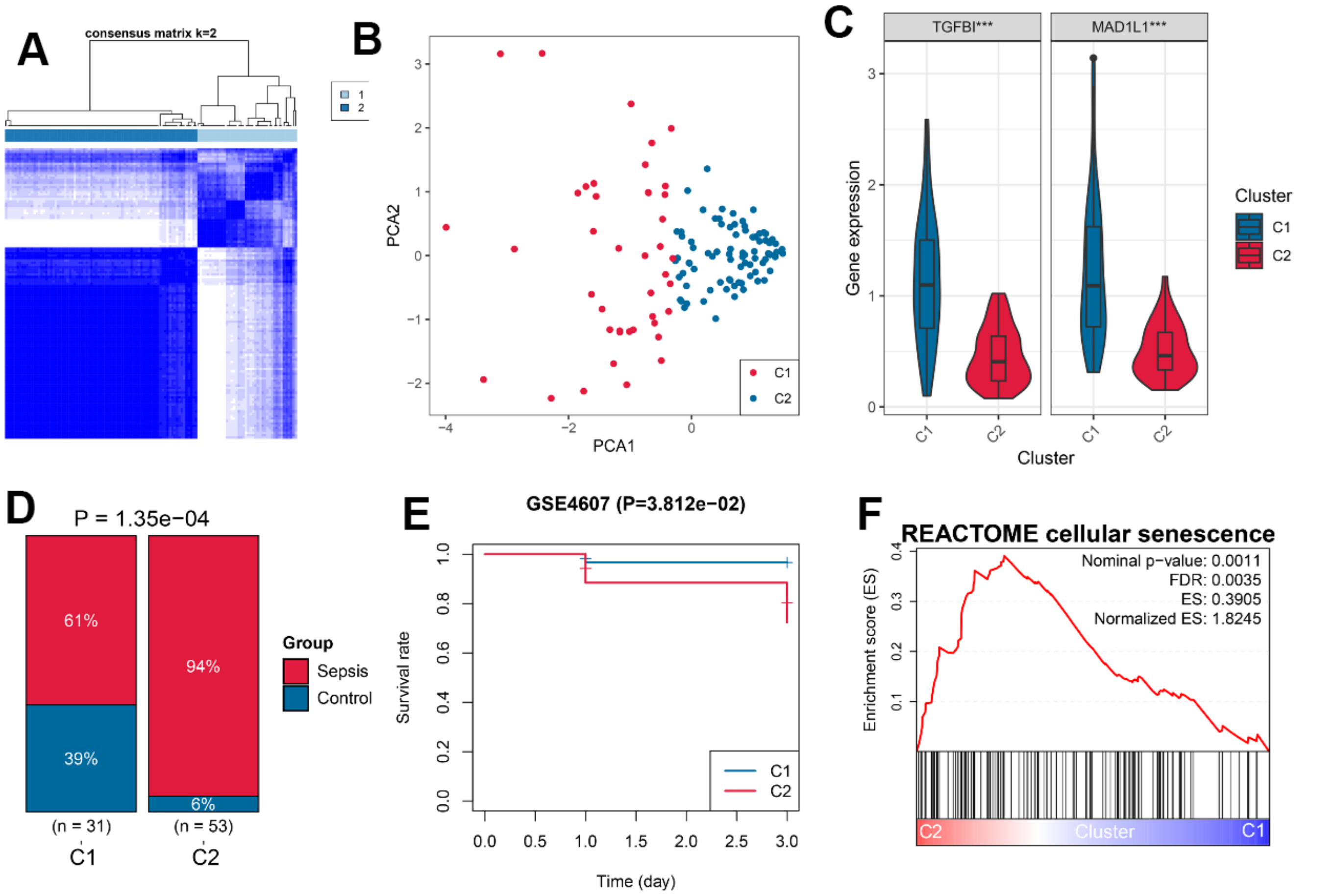

Supplementary Figure 1. Unsupervised clustering analysis of TGFBI and MAD1L1 gene expressions in the GSE4607 cohort. (**A**) Consensus clustering was conducted to classify subjects into C1 and C2 subgroups. (**B**) PCA was performed to validate the clustering results. (**C**) The expression levels of TGFBI and MAD1L1 were significantly downregulated in the C2 subgroup. (**D**) The clustering analysis was associated with sepsis characteristics. (**E**) Sepsis patients in the C2 subgroup had unfavorable clinical outcomes. (**F**) The C2 subgroup exhibited higher levels of cellular senescence. Abbreviations: C1, cluster 1; C2, cluster 2; PCA, principal component analysis; ***P < 0.001.

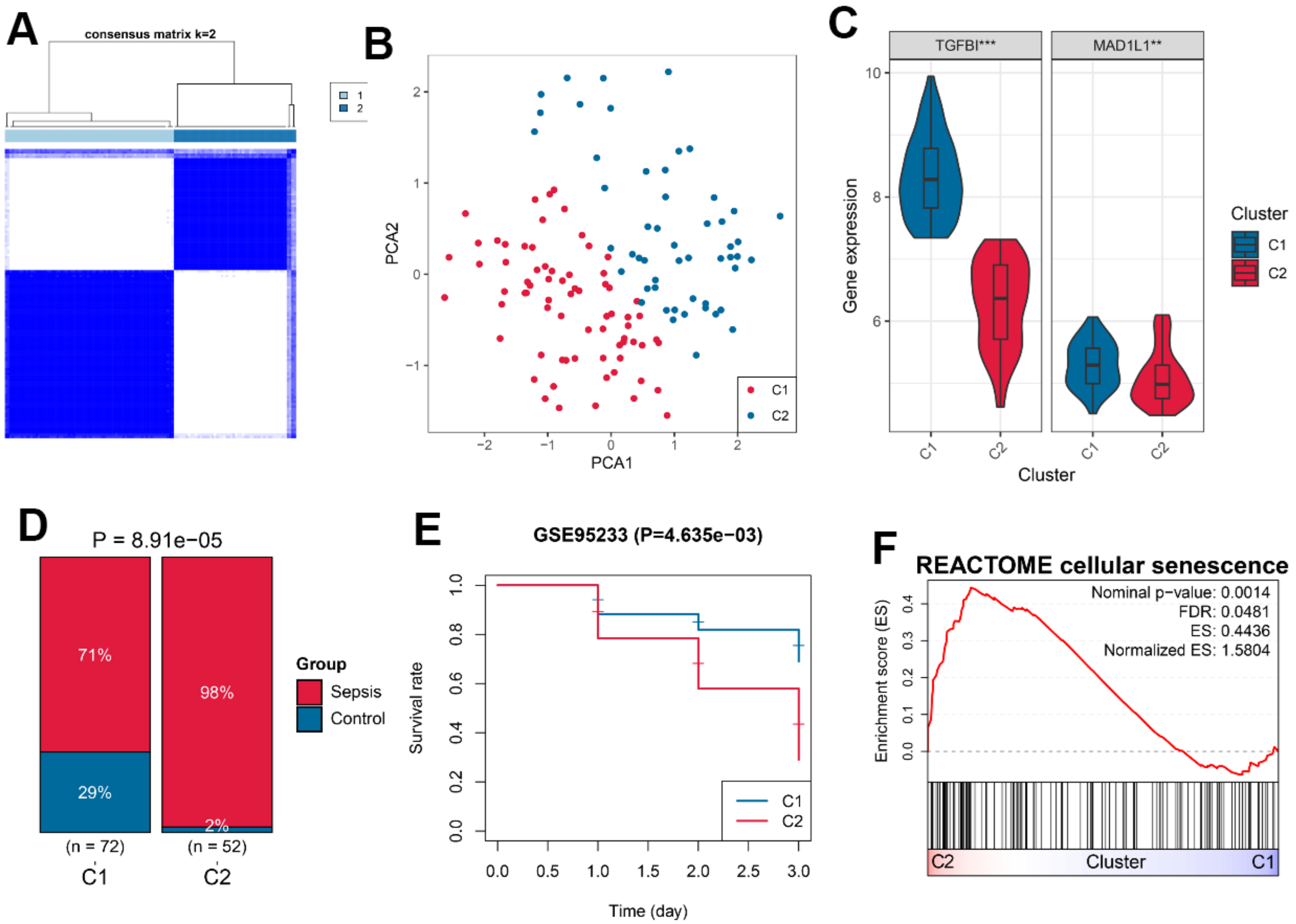

Supplementary Figure 2. Unsupervised clustering analysis of TGFBI and MAD1L1 gene expressions in the GSE95233 cohort. (**A**) Consensus clustering was conducted to classify subjects into C1 and C2 subgroups. (**B**) PCA was performed to validate the clustering results. (**C**) The expression levels of TGFBI and MAD1L1 were significantly downregulated in the C2 subgroup. (**D**) The clustering analysis was associated with sepsis characteristics. (**E**) Sepsis patients in the C2 subgroup had unfavorable clinical outcomes. (**F**) The C2 subgroup exhibited higher levels of cellular senescence. **P < 0.01; ***P < 0.001.
