# Supplementary material for: Unlocking the potential of senescence-related gene signature as a diagnostic and prognostic biomarker in sepsis: insights from meta-analyses, single-cell RNA sequencing, and in vitro experiments
Source: Aging (Albany NY). 2024 Feb 26;16(4):3989–4013. doi: 10.18632/aging.205574 (PMC10929830; doi:10.18632/aging.205574)
Supplement: Supplementary Table 1 [file aging-16-205574-s003.docx]

Supplementary Table 1. **866 senescence-related genes collected from the CellAge.**

| **Gene symbol** |
| --- |
| AAK1, ABCB1, ABCC6, ABI3, ABI3BP, ACER2, ACKR1, ACLY, ADCK5, AGO2, AGR2, AGT, AHR, AKAP4, AKR1B1, AKT1, AKT1S1, AKT3, AKTIP, ALDH2, ALDOA, ALKBH3, ALOX15B, ANAPC1, ANLN, ANXA5, APEX1, AR, ARF1, ARG2, ARID1A, ARID1B, ARID3A, ARID4B, ARPC1B, ARRB1, ASAH1, ASF1A, ASPH, ASXL2, ATF3, ATF6, ATF7IP, ATG10, ATG12, ATG16L1, ATG4D, ATG5, ATG7, ATM, ATP6V0A2, ATP6V0C, ATR, ATRAID, ATRX, ATXN10, AURKA, AURKB, AXL, BAG3, BAP1, BAZ1A, BCL11B, BCL2, BCL2L1, BCL2L2, BCL3, BCL6, BCLAF1, BECN1, BHLHE40, BIN1, BIRC5, BLK, BLVRA, BMI1, BMP4, BMPR2, BMS1, BNIP3L, BRAF, BRCA1, BRD4, BRD7, BRIP1, BTG1, BTG2, BTG3, BUB1, BUB1B, CALR, CAPNS1, CARF, CARM1, CASP2, CAV1, CAVIN1, CBS, CBX5, CBX7, CBX8, CCL2, CCN2, CCN6, CCNA2, CCNB1, CCND1, CCND3, CD28, CD34, CD40LG, CD82, CDC25A, CDC45, CDC6, CDC7, CDCA2, CDCA4, CDH1, CDK1, CDK18, CDK2, CDK2AP1, CDK4, CDK5, CDK5R1, CDK6, CDKN1A, CDKN1B, CDKN1C, CDKN2A, CDKN2AIP, CDKN2B, CEACAM1, CEBPB, CEBPG, CENPA, CHAF1B, CHD5, CHEK1, CHEK2, CHUK, CIP2A, CIT, CKAP2, CKB, CLCA2, CLPP, CLSPN, CLU, CNOT6, CNOT6L, COX5B, CPEB1, CPT1C, CREG1, CRISPLD2, CSNK1A1, CSNK2A1, CTH, CTNNAL1, CTNNB1, CTSB, CTSD, CUL4B, CUX1, CXCL1, CXCR2, CYB5R3, CYBB, CYP26A1, DAO, DDAH2, DDB1, DDB2, DDIT4, DEK, DEPTOR, DGCR8, DHCR24, DHRS2, DHX9, DICER1, DIDO1, DIRAS3, DKC1, DLC1, DMTF1, DNMT1, DNMT3A, DNMT3B, DOT1L, DPP4, DPY30, DTL, DUSP1, DUSP16, DUSP21, DUSP3, DUSP6, DYRK1A, E2F1, E2F3, E2F7, ECT2, EEF1E1, EGFR, EGLN1, EGR2, EHF, EHMT2, EID3, EIF2AK2, EIF2AK3, EIF4E, EIF4EBP1, EIF4G2, ELAVL1, ELOA, ENDOG, ENG, ENO1, ENTPD7, EP300, EPAS1, EPHA3, EPHA5, EPOR, ERBB2, ERCC1, ERRFI1, ERVW-1, ESPL1, ESR1, ESRRB, ETS1, ETS2, ETV6, EWSR1, EZH2, FANCD2, FASN, FASTK, FBXO22, FBXO31, FBXO4, FBXO5, FBXW11, FDPS, FERMT1, FERMT2, FGF21, FGFR1, FGFR2, FGFR3, FIS1, FLT1, FNTB, FOS, FOXA1, FOXD1, FOXM1, FOXO1, FOXO3, FOXO4, FOXP1, FOXP3, FOXQ1, FSCN1, FXN, FXR1, G6PD, GADD45G, GAPDH, GATA4, GDF15, GEMIN2, GGCT, GJA1, GKN1, GLI1, GMNN, GMPS, GNG11, GNMT, GPC3, GRIK2, GRK4, GRK6, GRN, GRPR, GRSF1, GSK3A, GSK3B, GTSE1, HAS1, HAUS4, HBP1, HDAC1, HDAC2, HDAC3, HDAC4, HDAC7, HELLS, HEPACAM, HIF1A, HIPK2, HIRA, HIVEP1, HJURP, HK3, HMGA1, HMGA2, HMGB1, HMGB2, HMGB3, HMGCR, HNRNPA1, HNRNPA3, HNRNPAB, HNRNPC, HNRNPD, HOPX, HOXA9, HRAS, HS2ST1, HSF1, HSP90AA1, HSP90AB1, HSPA1A, HSPA2, HSPA5, HSPA9, HSPB1, HTRA1, HYOU1, ID1, IFI16, IFNG, IGFBP1, IGFBP3, IGFBP5, IGFBP7, IKBKG, IL1A, IL1R1, IL1RN, IL24, IL6, ILF3, ILK, IMMT, INCENP, ING1, ING2, ING5, INPP4B, IRAK4, IRF1, IRF3, IRF5, IRF7, ITGB3, ITGB4, ITPK1, ITPKB, ITPR1, ITPR2, ITPR3, ITSN2, JAK2, JPT1, JUN, JUNB, KAT5, KCNA1, KCNJ12, KDM1A, KDM2B, KDM4A, KDM4C, KDM5B, KDM6B, KDR, KEAP1, KIF11, KIF20A, KIF2C, KIFC1, KL, KLF4, KNDC1, KRT19, KSR2, LAMP2, LATS1, LATS2, LAYN, LBR, LCN2, LEO1, LGALS3, LIMA1, LIMK1, LIN52, LIN54, LIN9, LMNA, LMNB1, LNCTAM34A, LOX, LOXL2, LPAR1, LPAR3, LRRK2, LY6D, MAD1L1, MAD2L1, MAEL, MAF, MAGEA2, MAGOHB, MAP2K1, MAP2K2, MAP2K3, MAP2K6, MAP2K7, MAP3K14, MAP3K5, MAP3K6, MAP3K7, MAP4K1, MAPK1, MAPK12, MAPK14, MAPK9, MAPKAPK5, MARCHF5, MARCKS, MAST1, MATK, MAVS, MCAM, MCL1, MCM3AP, MCM7, MCRS1, MCU, MDC1, MDH1, MDK, MDM2, MDP1, ME1, ME2, MECP2, MED12, MEF2A, MEIS2, MEN1, MEOX1, MET, METTL14, MIF, MINK1, MITF, MKRN1, MME, MMP7, MMP9, MNX1, MOB3A, MORC3, MORF4L1, MOS, MRTFA, MRTFB, MSN, MST1, MT1G, MTDH, MTHFD2, MTOR, MUC4, MUS81, MVK, MVP, MXD4, MYBBP1A, MYBL2, MYC, MYCN, MYD88, MYLK, NACC1, NADK, NAMPT, NANOG, NBN, NBR1, NCAPG2, NCAPH2, NDRG1, NDST2, NEDD4, NEK1, NEK2, NEK4, NEK6, NEK9, NF1, NF2, NFE2L2, NFKB2, NFKBIA, NHEJ1, NINJ1, NIPA2, NLK, NLRX1, NOLC1, NOTCH1, NOTCH3, NOX1, NOX4, NPM1, NQO1, NR1H2, NR2E1, NRAS, NRF1, NRSN2, NSUN2, NTN4, NUAK1, NUAK2, NUDT5, NUTF2, OGG1, OGT, OPA1, ORAI1, ORC1, OTX2, OXTR, P2RY14, P3H1, PAK2, PAK4, PAPSS2, PARK7, PARP1, PATZ1, PAX8, PBRM1, PCGF2, PDCD10, PDCD11, PDCD4, PDGFB, PDIK1L, PDPK1, PDZD2, PEA15, PEBP1, PELP1, PES1, PEX19, PGR, PHB, PHB2, PHGDH, PI4KB, PIK3C2A, PIK3CA, PIK3R5, PIM1, PIN1, PINK1, PINX1, PIR, PITX1, PLA2G2A, PLA2R1, PLD2, PLK1, PML, PMVK, PNPT1, PON1, POT1, POU3F1, POU5F1, PPARG, PPARGC1A, PPIB, PPM1B, PPM1D, PPP1R13B, PPP2R1A, PPP2R5A, PRKAA2, PRKCD, PRKCH, PRKD1, PRKD2, PRKDC, PRKN, PRMT1, PRMT6, PRODH, PROX1, PRPF19, PSMA2, PSMA5, PSMB1, PSMB5, PSMD14, PTEN, PTGS2, PTK2, PTPN1, PTPN6, PTTG1, PURB, PYGL, RACGAP1, RACK1, RAD21, RAD23B, RAD51C, RAF1, RAN, RANBP9, RAP1GAP, RAPGEF4, RARB, RASSF1, RASSF4, RASSF5, RB1, RBBP4, RBL1, RBL2, RBM38, RBM39, RBP1, RBP2, RBPJ, RBX1, RCC1, RECK, RECQL4, RELA, RELB, RHOA, RNASEH2A, RNASEH2B, RNASEL, ROMO1, RPL11, RPS14, RPS6KB1, RPS9, RPTOR, RRAD, RRAS2, RRM1, RRM2, RRM2B, RRP8, RTN4, RUNX1, RUVBL2, S100A6, SALL1, SAMHD1, SAT2, SDC1, SELENBP1, SELENOH, SENP1, SENP7, SERPINB2, SERPINE1, SETD1A, SFN, SFRP1, SGK1, SIAH1, SIK1, SIN3B, SIRT1, SIRT2, SIRT3, SIRT6, SIRT7, SIX1, SIX6, SKP2, SLC13A3, SLC16A7, SLC25A5, SLC31A2, SLC52A1, SLC5A2, SMAD1, SMAD2, SMAD3, SMAD5, SMAD6, SMARCA2, SMARCA4, SMARCB1, SMARCD1, SMC1A, SMC2, SMG1, SMURF2, SNAI1, SOCS1, SOD1, SOD2, SORBS2, SOX1, SOX2, SOX4, SOX5, SP1, SPAG9, SPARC, SPHK1, SPI1, SPIN1, SPOP, SRC, SREBF1, SRF, SRSF1, SRSF2, SRSF3, SSX2, STAG2, STAT1, STAT3, STAT5A, STAT5B, STAT6, STIM1, STK32C, STK4, STK40, STN1, STUB1, SUMO2, SUMO3, SUPT5H, SUV39H1, SUZ12, SYK, SYT1, SYT7, TACC3, TAGLN, TBK1, TBPL1, TBX2, TBX3, TEAD1, TEAD4, TERF2, TERT, TFDP1, TFG, TGFB1I1, TGFB2, TGFBI, TGFBR1, TGFBR2, THRB, TIGAR, TIMELESS, TLR10, TLR2, TLR3, TLR4, TLR8, TMEM9B, TNFSF13, TNFSF15, TOM1, TOP1, TOP3A, TOPBP1, TP53, TP53BP1, TP53BP2, TP53I3, TP53INP1, TP63, TPP1, TPR, TPX2, TRA2B, TRDMT1, TRIM28, TRPM7, TRPM8, TRRAP, TSC22D1, TTK, TWIST1, TWIST2, TXN, TXNIP, TYK2, TYMS, UBE2C, UBE2I, UBE2N, UBE2V1, UBE2V2, UBE3A, UBTD1, UHRF1, ULK3, USP1, USP28, UTP6, VCAN, VDR, VEGFA, VENTX, WEE1, WIF1, WIPI1, WNT16, WNT2, WNT5A, WNT7B, WRN, WSB1, WT1, WWP1, XAF1, XIAP, XPC, XPO1, YAP1, YBX1, YEATS4, YPEL3, YWHAB, YWHAZ, YY1, ZCCHC10, ZDHHC3, ZEB1, ZFP36, ZFX, ZMAT3, ZMPSTE24, ZMYND11, ZNF148, ZNF207, ZNF217. |
