# Supplementary material for: Unlocking the potential of senescence-related gene signature as a diagnostic and prognostic biomarker in sepsis: insights from meta-analyses, single-cell RNA sequencing, and in vitro experiments
Source: Aging (Albany NY). 2024 Feb 26;16(4):3989–4013. doi: 10.18632/aging.205574 (PMC10929830; doi:10.18632/aging.205574)
Supplement: Supplementary Tables 2 and 3 [file aging-16-205574-s004.pdf]

Supplementary Table 2. The detailed information of the datasets downloaded from the GEO.

ID	Platform	Experimental type	Tissue	Sample size (control/sepsis)	Region	PMID
GSE4607	GPL570	Microarray	Whole blood	15/69	USA	17374846
GSE9692	GPL570	Microarray	Whole blood	15/30	USA	18460642
GSE13904	GPL570	Microarray	Whole blood	18/158	USA	19325468
GSE26378	GPL570	Microarray	Whole blood	21/82	USA	21738952
GSE26440	GPL570	Microarray	Whole blood	32/98	USA	19624809
GSE28750	GPL570	Microarray	Whole blood	20/10	Australia	21682927
GSE54514	GPL6947	Microarray	Whole blood	18/145	Australia	23807251
GSE57065	GPL570	Microarray	Whole blood	25/82	France	30671061
GSE65682	GPL13667	Microarray	Whole blood	42/760	Malta	26956172
GSE67652	GPL16699	Microarray	Whole blood	12/12	Brazil	26047321
GSE69063	GPL19983	Microarray	Whole blood	33/57	Australia	NA
GSE69528	GPL10558	Microarray	Whole blood	55/83	USA	19903332
GSE95233	GPL570	Microarray	Whole blood	22/102	France	30671061
GSE131761	GPL13497	Microarray	Whole blood	15/81	Spain	34144116
GSE175453	GPL18573<br>GPL24676	Single-cell RNA-seq	Whole blood	5/4	USA	34484194

Supplementary Table 3 The primer sequence adopted in the RT-qPCR experiments.

ID	Sequence (5'-3')
TGFBI-F	CACTCTCAAACCTTTACGAGACC
TGFBI-R	CGTTGCTAGGGGCGAAGATG
MAD1L1-F	TGGACTGGATATTTCTACCTCGG
MAD1L1-R	CCTCACGCTCGTAGTTCCTG
GAPDH-F	GGAGCGAGATCCCTCCAAAAT
GAPDH-R	GGCTGTTGTCATACTTCTCATGG
