# Supplementary material for: Unlocking the potential of senescence-related gene signature as a diagnostic and prognostic biomarker in sepsis: insights from meta-analyses, single-cell RNA sequencing, and in vitro experiments
Source: Aging (Albany NY). 2024 Feb 26;16(4):3989–4013. doi: 10.18632/aging.205574 (PMC10929830; doi:10.18632/aging.205574)
Supplement: Supplementary Table 4 [file aging-16-205574-s005.docx]

Supplementary Table 4. **80 senescence-related genes were differentially expressed between control and sepsis samples.**

| **Gene symbol** | **logFC** | **P-value** | **FDR** | **Senescence type** | **Senescence effect** | **Reference PMID** |
| --- | --- | --- | --- | --- | --- | --- |
| TXN | 1.775072 | 1.90E-80 | 1.23E-77 | Unclear | Inhibits | 20074557 |
| ANAPC1 | -1.397366 | 4.43E-69 | 7.20E-67 | Unclear | Inhibits | 25255445 |
| PARP1 | -1.427137 | 9.88E-68 | 1.29E-65 | Unclear | Inhibits | 29590171 |
| INPP4B | -1.085335 | 1.14E-65 | 1.24E-63 | Unclear | Inhibits | 19647222 |
| SMAD3 | -1.568316 | 3.54E-65 | 3.29E-63 | Stress-induced | Induces | 27892764 |
| BCL11B | -3.270284 | 2.71E-64 | 2.21E-62 | Unclear | Inhibits | 26096706 |
| BCL2 | -1.046673 | 1.36E-63 | 9.84E-62 | Oncogene-induced | Induces | 12670482 |
| MAPK14 | 1.891386 | 6.51E-63 | 4.24E-61 | Replicative | Induces | 12581156 |
| DPP4 | -1.644954 | 3.61E-62 | 2.14E-60 | Oncogene-induced | Induces | 29481642 |
| HK3 | 2.388338 | 1.30E-61 | 7.05E-60 | Unclear | Induces | 26583757 |
| MMP9 | 3.015026 | 1.54E-61 | 7.73E-60 | Unclear | Inhibits | 17510426 |
| PRPF19 | -1.480659 | 1.76E-60 | 8.18E-59 | Replicative | Inhibits | 16388800 |
| DNMT1 | -1.629842 | 2.18E-59 | 9.44E-58 | Unclear | Inhibits | 21572997 |
| ZDHHC3 | 1.272459 | 2.34E-58 | 9.51E-57 | Stress-induced | Inhibits | 29055014 |
| NOLC1 | -1.212349 | 3.08E-58 | 1.18E-56 | Unclear | Induces | 28493459 |
| ETS1 | -1.830329 | 8.53E-54 | 2.41E-52 | Oncogene-induced | Induces | 11234019 |
| MATK | -1.675258 | 1.64E-53 | 4.44E-52 | Unclear | Induces | 26583757 |
| BIN1 | -1.505503 | 3.15E-53 | 8.21E-52 | Oncogene-induced | Induces | 18267069 |
| PRKCH | -2.095535 | 6.28E-53 | 1.57E-51 | Stress-induced | Induces | 25412309 |
| DKC1 | -1.445015 | 7.37E-53 | 1.78E-51 | Replicative | Inhibits | 25732822 |
| PEBP1 | -1.985757 | 4.12E-51 | 9.25E-50 | Stress-induced | Induces | 23814485 |
| HSPA9 | -1.394722 | 2.82E-48 | 5.74E-47 | Stress-induced | Induces | 17306926 |
| HMGB2 | 1.492625 | 2.05E-44 | 3.93E-43 | Oncogene-induced | Inhibits | 27799366 |
| ATM | -1.054076 | 2.23E-44 | 4.15E-43 | Replicative | Inhibits | 9312059 |
| CDKN1C | -1.973955 | 3.08E-44 | 5.57E-43 | Unclear | Induces | 22705236 |
| NF2 | -1.103484 | 6.64E-44 | 1.17E-42 | Oncogene-induced | Induces | 18267069 |
| DDAH2 | 1.913922 | 1.50E-43 | 2.51E-42 | Unclear | Inhibits | 24764313 |
| TRRAP | -1.061763 | 1.99E-43 | 3.16E-42 | Oncogene-induced | Inhibits | 28806777 |
| LMNB1 | 1.571971 | 8.03E-43 | 1.22E-41 | Oncogene-induced | Inhibits | 22155925 |
| PDCD4 | -1.480945 | 1.60E-42 | 2.37E-41 | Replicative | Inhibits | 30687637 |
| AKR1B1 | -1.265349 | 2.13E-41 | 3.02E-40 | Stress-induced | Inhibits | 21182935 |
| HNRNPA1 | -1.367364 | 9.17E-41 | 1.27E-39 | Unclear | Inhibits | 31257225 |
| IMMT | -1.142111 | 9.41E-41 | 1.28E-39 | Unclear | Inhibits | 32264951 |
| IL1R1 | 2.01929 | 9.90E-40 | 1.32E-38 | Oncogene-induced | Induces | 18267069 |
| MAD1L1 | -1.591548 | 2.09E-39 | 2.72E-38 | Stress-induced | Induces | 19766114 |
| RBBP4 | -1.101301 | 3.43E-39 | 4.38E-38 | Unclear | Inhibits | 26491019 |
| RNASEH2B | -1.248698 | 6.29E-39 | 7.87E-38 | Stress-induced | Inhibits | 30532030 |
| ZMYND11 | -1.69141 | 2.46E-38 | 2.91E-37 | Unclear | Inhibits | 17721438 |
| PEA15 | -1.019387 | 7.29E-38 | 8.33E-37 | Replicative | Induces | 18267069 |
| KDM1A | -1.008928 | 1.67E-37 | 1.84E-36 | Oncogene-induced | Inhibits | 29438700 |
| MAF | -1.520739 | 1.47E-36 | 1.57E-35 | Stress-induced | Inhibits | 26496036 |
| TRIM28 | -1.02252 | 1.54E-35 | 1.54E-34 | Oncogene-induced | Induces | 25160591 |
| SGK1 | -2.026928 | 2.54E-34 | 2.33E-33 | Replicative | Inhibits | 26230157 |
| MAP2K6 | 1.5838 | 4.80E-34 | 4.34E-33 | Unclear | Induces | 12208764 |
| PYGL | 1.196569 | 4.66E-33 | 4.16E-32 | Unclear | Inhibits | 23177934 |
| NPM1 | -1.365964 | 9.79E-33 | 8.50E-32 | Oncogene-induced | Inhibits | 23536448 |
| PDCD10 | 1.643584 | 2.59E-32 | 2.22E-31 | Replicative | Induces | 25655101 |
| RBX1 | 1.552741 | 5.92E-32 | 5.00E-31 | Unclear | Inhibits | 19509229 |
| ABI3 | -1.28939 | 9.28E-31 | 7.55E-30 | Oncogene-induced | Induces | 21223585 |
| MYC | -1.958506 | 1.94E-30 | 1.56E-29 | Oncogene-induced | Inhibits | 16537449 |
| RRAS2 | -1.358122 | 2.37E-29 | 1.82E-28 | Unclear | Inhibits | 29440281 |
| APEX1 | -1.311967 | 9.81E-29 | 7.42E-28 | Replicative | Inhibits | 29750271 |
| LCN2 | 3.690423 | 1.15E-27 | 8.04E-27 | Stress-induced | Inhibits | 24452457 |
| ASPH | 1.265133 | 7.32E-27 | 5.07E-26 | Unclear | Inhibits | 26683595 |
| BCL6 | 1.220246 | 7.81E-27 | 5.35E-26 | Replicative | Inhibits | 11914273 |
| WIPI1 | 1.312094 | 3.04E-26 | 2.04E-25 | Unclear | Inhibits | 30352807 |
| CBX7 | -1.380073 | 3.56E-26 | 2.37E-25 | Replicative | Inhibits | 14647293 |
| TP53I3 | 1.845056 | 6.79E-26 | 4.46E-25 | Replicative | Inhibits | 28259183 |
| HAUS4 | 1.083045 | 9.17E-26 | 5.97E-25 | Unclear | Inhibits | 32264951 |
| CTSD | 1.030938 | 3.31E-24 | 2.01E-23 | Unclear | Inhibits | 26657266 |
| CREG1 | 1.226196 | 3.73E-23 | 2.15E-22 | Oncogene-induced | Induces | 21263217 |
| CEACAM1 | 2.234437 | 7.74E-23 | 4.42E-22 | Stress-induced | Induces | 23552604 |
| MEF2A | 1.146717 | 1.45E-21 | 7.57E-21 | Replicative | Inhibits | 31182679 |
| WSB1 | 1.172718 | 1.95E-21 | 1.01E-20 | Oncogene-induced | Inhibits | 27958289 |
| DUSP6 | -1.306915 | 3.90E-21 | 2.00E-20 | Oncogene-induced | Inhibits | 25499223 |
| HSP90AB1 | -1.222644 | 7.17E-20 | 3.43E-19 | Oncogene-induced | Inhibits | 27793846 |
| IL1RN | 1.285776 | 2.07E-19 | 9.75E-19 | Replicative | Unclear | 12764021 |
| ETS2 | 1.170285 | 4.07E-19 | 1.90E-18 | Oncogene-induced | Induces | 11234019 |
| CLU | 1.34746 | 7.10E-17 | 2.89E-16 | Stress-induced | Inhibits | 31376806 |
| TGFBI | -1.604083 | 1.16E-16 | 4.59E-16 | Replicative | Induces | 22695319 |
| TLR2 | 1.004571 | 4.32E-16 | 1.66E-15 | Oncogene-induced | Induces | 31183403 |
| JAK2 | 1.095875 | 6.21E-16 | 2.36E-15 | Unclear | Induces | 24681574 |
| HOPX | -1.218411 | 1.02E-15 | 3.81E-15 | Oncogene-induced | Induces | 25345926 |
| SERPINB2 | 1.117892 | 1.15E-12 | 3.66E-12 | Unclear | Induces | 28794016 |
| TFDP1 | 1.112903 | 1.16E-11 | 3.60E-11 | Unclear | Inhibits | 22012588 |
| GNG11 | 1.005759 | 6.15E-11 | 1.76E-10 | Stress-induced | Induces | 17092487 |
| MARCKS | 1.026415 | 1.52E-10 | 4.20E-10 | Unclear | Induces | 22619307 |
| CTNNAL1 | 1.073454 | 1.11E-09 | 2.89E-09 | Oncogene-induced | Inhibits | 21278790 |
| RAP1GAP | 1.298989 | 3.49E-06 | 7.21E-06 | Oncogene-induced | Induces | 18267069 |
| XAF1 | -1.017942 | 1.18E-05 | 2.28E-05 | Stress-induced | Induces | 26802028 |
