# Supplementary material for: Unlocking the potential of senescence-related gene signature as a diagnostic and prognostic biomarker in sepsis: insights from meta-analyses, single-cell RNA sequencing, and in vitro experiments
Source: Aging (Albany NY). 2024 Feb 26;16(4):3989–4013. doi: 10.18632/aging.205574 (PMC10929830; doi:10.18632/aging.205574)
Supplement: Supplementary Tables 5 and 6 [file aging-16-205574-s006.pdf]

Supplementary Table 5 The coefficients of the variables in the LASSO regression model.

Variable	Coefficients
MAPK14	-0.045773175
DPP4	-0.101958152
BIN1	-0.060200161
ATM	-0.375968363
NF2	-0.021334301
MAD1L1	-0.086230383
SGK1	-0.017652457
ABI3	-0.047358246
APEX1	0.185429514
ASPH	0.215059072
HAUS4	-0.226662103
TGFBI	-0.094521895
GNG11	-0.00914029
RAP1GAP	0.083434285
XAF1	-0.04001422

Supplementary Table 6. Univariate Cox regression analyses of the 80 differentially-expressed genes.

Gene symbol	HR	HR.95L	HR.95H	P value
ATM	0.39366175	0.246367289	0.629018463	9.67E-05
TGFBI	0.730466464	0.616705515	0.865212393	0.000276745
RAP1GAP	1.178497992	1.074182833	1.29294332	0.000514128
MAD1L1	0.605689585	0.447387666	0.820004444	0.001179219
CTNNAL1	1.27077576	1.094948094	1.474837976	0.001611758
RBX1	1.47202775	1.153470599	1.878561706	0.001887033
ABI3	0.637589229	0.478212199	0.850082924	0.002164388
BIN1	0.604028143	0.431088347	0.846346231	0.003396944
PRKCH	0.693931582	0.540413655	0.891060091	0.004181953
DPP4	0.570700246	0.385879889	0.844041836	0.00496727
NF2	0.569812362	0.37991762	0.854622452	0.006537019
SGK1	0.765133269	0.629911394	0.929382965	0.006974638
BCL11B	0.779756763	0.646484066	0.940503628	0.009284823
PEA15	0.582643242	0.386353911	0.8786585	0.009962868
HAUS4	0.686259756	0.513934592	0.916366519	0.010711952
ASPH	1.345178548	1.059741501	1.707496898	0.01481775
PYGL	0.745420746	0.584120723	0.951262414	0.018197302
MATK	0.637663283	0.43867229	0.926920781	0.018393858
PDCD4	0.706718843	0.522917016	0.955125781	0.023901222
ZDHHC3	1.587360197	1.057863253	2.381888572	0.025639065
XAF1	0.872718502	0.770351637	0.988688213	0.032462005
INPP4B	0.486382051	0.250026236	0.946170706	0.033758833
MAPK14	0.756311711	0.583136287	0.980915468	0.035273761
SMAD3	0.668593055	0.457181838	0.977765597	0.037902281
BCL6	0.77130258	0.601690124	0.988727662	0.040419687
CREG1	1.280311446	1.005672111	1.629952128	0.044869306
IMMT	0.693134953	0.482920175	0.994856062	0.046818887
TRIM28	0.681633638	0.459004202	1.012244363	0.057479942
PDCD10	1.247377977	0.992096926	1.568346577	0.058484581
CDKN1C	0.805332466	0.642608885	1.009261459	0.060119672
LMNB1	0.774533249	0.591071549	1.014939316	0.063961444
TFDP1	1.159473081	0.980305456	1.371386659	0.084040634
LCN2	1.079240051	0.978984373	1.189762697	0.125279024
TLR2	0.835564342	0.663439607	1.052345628	0.126900035
CBX7	0.838713503	0.668722698	1.051916351	0.12801142
PRPF19	0.759320726	0.530242029	1.087367528	0.132892489
DUSP6	0.842992067	0.67452229	1.053539126	0.133233547
WIPI1	1.189246342	0.932279094	1.517042344	0.162894758
HMGB2	1.213388717	0.911967818	1.614434357	0.184344011
MEF2A	0.851089935	0.66912373	1.082541309	0.188932257
RNASEH2B	0.807465289	0.586671289	1.11135521	0.18946809
MYC	0.886492024	0.738822913	1.063675874	0.194987837
PARP1	0.76342774	0.505183501	1.153683588	0.200069767
ANAPC1	0.751821004	0.480752215	1.175730042	0.211168358
BCL2	0.678566871	0.36869976	1.248856246	0.212788969
SERPINB2	1.10873739	0.935780401	1.313661408	0.232909204
ETS1	0.850532971	0.645196594	1.121218466	0.250817732

TRRAP	0.788888305	0.518670832	1.199883855	0.26773657
IL1RN	0.88898152	0.719914958	1.097752079	0.274216537
ZMYND11	0.881736392	0.695457627	1.117910041	0.298595848
PEBP1	0.878558203	0.682503546	1.130931144	0.314924223
KDM1A	0.821246741	0.557176374	1.210471659	0.319765173
APEX1	1.126842604	0.871086918	1.4576895	0.363244788
CLU	0.91415635	0.751788238	1.111592055	0.368333367
DDAH2	1.101039762	0.885001206	1.369815711	0.387737997
HK3	0.911372481	0.736298466	1.128074875	0.393833377
HOPX	0.930361458	0.767205359	1.128214802	0.463121263
TP53I3	1.060923394	0.904304437	1.244667615	0.468036685
RBBP4	0.874829864	0.608385996	1.257963359	0.470543171
MMP9	0.943256717	0.803559471	1.107240055	0.475035452
GNG11	0.93485309	0.776351325	1.125714958	0.477277329
JAK2	0.927659388	0.7499593	1.147464856	0.488865986
MAP2K6	0.931972614	0.747000176	1.16274799	0.532536848
ETS2	1.071162987	0.855832424	1.340671507	0.548261987
IL1R1	1.057677778	0.865394254	1.292685129	0.583854462
TXN	1.092893642	0.765257503	1.560803401	0.625168271
MAF	0.931040408	0.696918121	1.243813606	0.628725756
HSPA9	0.923509329	0.664050175	1.284344938	0.636307179
CTSD	1.064994322	0.809977853	1.400301132	0.652065537
RRAS2	0.942215841	0.719416571	1.234014792	0.665450782
DKC1	0.935647333	0.670873155	1.304920201	0.695129076
HSP90AB1	1.041049273	0.83982119	1.290493263	0.713557498
NOLC1	0.932974854	0.606904598	1.434232138	0.751835916
DNMT1	0.951891318	0.694466622	1.30473813	0.759240039
WSB1	1.038282766	0.816365575	1.320524941	0.759442773
NPM1	1.041388863	0.798799962	1.357649995	0.764387513
HNRNPA1	0.957813804	0.71382272	1.285203254	0.773865589
MARCKS	1.020786772	0.842575294	1.236691417	0.833538247
CEACAM1	1.007323368	0.881865964	1.150628791	0.914377669
AKR1B1	0.987513762	0.716213616	1.361581807	0.938887743
