# Supplementary material for: Unlocking the potential of senescence-related gene signature as a diagnostic and prognostic biomarker in sepsis: insights from meta-analyses, single-cell RNA sequencing, and in vitro experiments
Source: Aging (Albany NY). 2024 Feb 26;16(4):3989–4013. doi: 10.18632/aging.205574 (PMC10929830; doi:10.18632/aging.205574)
Supplement: Supplementary Table 7 [file aging-16-205574-s007.docx]

Supplementary Table 7. **The sample ID of the cases in C1 and C2 subgroups.**

| **Cohort** | **Cluster** | **Sample ID** |
| --- | --- | --- |
| GSE65682 | C1 | GSM1602801, GSM1602802, GSM1602803, GSM1602804, GSM1602806, GSM1602807, GSM1602808, GSM1602809, GSM1602810, GSM1602812, GSM1602814, GSM1602816, GSM1602820, GSM1602823, GSM1602825, GSM1602827, GSM1602828, GSM1602829, GSM1602830, GSM1602831, GSM1602832, GSM1602834, GSM1602835, GSM1602836, GSM1602837, GSM1602838, GSM1602846, GSM1602849, GSM1602850, GSM1602853, GSM1602856, GSM1602861, GSM1602862, GSM1602863, GSM1602864, GSM1602870, GSM1602871, GSM1602872, GSM1602874, GSM1602875, GSM1602876, GSM1602881, GSM1602882, GSM1602884, GSM1602886, GSM1602888, GSM1602891, GSM1602892, GSM1602893, GSM1602894, GSM1602895, GSM1602896, GSM1602897, GSM1602898, GSM1602899, GSM1602900, GSM1602901, GSM1602904, GSM1602905, GSM1602906, GSM1602907, GSM1602909, GSM1602910, GSM1602911, GSM1602912, GSM1602913, GSM1602914, GSM1602915, GSM1602916, GSM1602917, GSM1602918, GSM1602919, GSM1602920, GSM1602921, GSM1602930, GSM1602938, GSM1602939, GSM1602941, GSM1602943, GSM1602944, GSM1602948, GSM1602949, GSM1602950, GSM1602951, GSM1602952, GSM1602954, GSM1602956, GSM1602957, GSM1602959, GSM1602960, GSM1602961, GSM1602962, GSM1602963, GSM1602964, GSM1602965, GSM1602966, GSM1602969, GSM1602970, GSM1602971, GSM1602972, GSM1691862, GSM1691864, GSM1691866, GSM1691867, GSM1691869, GSM1691870, GSM1691873, GSM1691875, GSM1691876, GSM1691878, GSM1691879, GSM1691881, GSM1691883, GSM1691884, GSM1691885, GSM1691887, GSM1691888, GSM1691889, GSM1691890, GSM1691891, GSM1691892, GSM1691894, GSM1691895, GSM1691899, GSM1691902, GSM1691904, GSM1691905, GSM1691906, GSM1691907, GSM1691911, GSM1691913, GSM1691914, GSM1691915, GSM1691917, GSM1691918, GSM1691919, GSM1691920, GSM1691921, GSM1691924, GSM1691926, GSM1691936, GSM1691938, GSM1691939, GSM1691940, GSM1691946, GSM1691947, GSM1691948, GSM1691950, GSM1691951, GSM1691952, GSM1691955, GSM1691957, GSM1691960, GSM1691962, GSM1691964, GSM1691965, GSM1691971, GSM1691974, GSM1691976, GSM1691982, GSM1691989, GSM1691991, GSM1691993, GSM1691997, GSM1691999, GSM1692000, GSM1692005, GSM1692010, GSM1692011, GSM1692015, GSM1692020, GSM1692025, GSM1692026, GSM1692027, GSM1692034, GSM1692035, GSM1692036, GSM1692041, GSM1692046, GSM1692048, GSM1692049, GSM1692051, GSM1692052, GSM1692053, GSM1692062, GSM1692066, GSM1692067, GSM1692068, GSM1692069, GSM1692074, GSM1692077, GSM1692078, GSM1692080, GSM1692082, GSM1692083, GSM1692085, GSM1692087, GSM1692089, GSM1692094, GSM1692095, GSM1692096, GSM1692101, GSM1692102, GSM1692104, GSM1692105, GSM1692106, GSM1692109, GSM1692112, GSM1692116, GSM1692120, GSM1692121, GSM1692125, GSM1692130, GSM1692131, GSM1692133, GSM1692142, GSM1692145, GSM1692147, GSM1692149, GSM1692150, GSM1692152, GSM1692154, GSM1692159, GSM1692161, GSM1692162, GSM1692163, GSM1692164, GSM1692167, GSM1692168, GSM1692173, GSM1692176, GSM1692179, GSM1692182, GSM1692185, GSM1692187, GSM1692189, GSM1692192, GSM1692193, GSM1692197, GSM1692199, GSM1692200, GSM1692202, GSM1692203, GSM1692204, GSM1692209, GSM1692210, GSM1692216, GSM1692218, GSM1692220, GSM1692222, GSM1692227, GSM1692229, GSM1692230, GSM1692231, GSM1692233, GSM1692237, GSM1692241, GSM1692242, GSM1692244, GSM1692245, GSM1692246, GSM1692247, GSM1692252, GSM1692253, GSM1692257, GSM1692259, GSM1692262, GSM1692263, GSM1692264, GSM1692265, GSM1692266, GSM1692270, GSM1692273, GSM1692274, GSM1692275, GSM1692276, GSM1692277, GSM1692280, GSM1692281, GSM1692282, GSM1692283, GSM1692286, GSM1692287, GSM1692291, GSM1692292, GSM1692293, GSM1692296, GSM1692297, GSM1692298, GSM1692300, GSM1692306, GSM1692311, GSM1692314, GSM1692315, GSM1692317, GSM1692318, GSM1692320, GSM1692321, GSM1692323, GSM1692324, GSM1692328, GSM1692331, GSM1692333, GSM1692334, GSM1692338, GSM1692340, GSM1692342, GSM1692344, GSM1692345, GSM1692346, GSM1692355, GSM1692360, GSM1692367, GSM1692368, GSM1692370, GSM1692371, GSM1692372, GSM1692373, GSM1692376, GSM1692377, GSM1692378, GSM1692380, GSM1692386, GSM1692389, GSM1692392, GSM1692394, GSM1692395, GSM1692396, GSM1692400, GSM1692402, GSM1692404, GSM1692405, GSM1692406, GSM1692407, GSM1692411, GSM1692413, GSM1692415, GSM1692417, GSM1692419, GSM1692420, GSM1692422, GSM1692423, GSM1692427, GSM1692429, GSM1692432, GSM1692433, GSM1692436, GSM1692437, GSM1692439, GSM1692442, GSM1692445, GSM1692451, GSM1692452, GSM1692455, GSM1692456, GSM1692460, GSM1692462, GSM1692466, GSM1692468, GSM1692469, GSM1692470, GSM1692472, GSM1692473, GSM1692475, GSM1692479, GSM1692480, GSM1692481, GSM1692483, GSM1692487, GSM1692488, GSM1692489, GSM1692491, GSM1692492, GSM1692493, GSM1692497, GSM1692498, GSM1692501, GSM1692502, GSM1692503, GSM1692504. |
|  | C2 | GSM1602805, GSM1602811, GSM1602813, GSM1602815, GSM1602817, GSM1602818, GSM1602819, GSM1602821, GSM1602822, GSM1602824, GSM1602826, GSM1602833, GSM1602839, GSM1602840, GSM1602841, GSM1602842, GSM1602843, GSM1602844, GSM1602845, GSM1602847, GSM1602848, GSM1602851, GSM1602852, GSM1602854, GSM1602855, GSM1602857, GSM1602858, GSM1602859, GSM1602860, GSM1602865, GSM1602866, GSM1602867, GSM1602868, GSM1602869, GSM1602873, GSM1602877, GSM1602878, GSM1602879, GSM1602880, GSM1602883, GSM1602885, GSM1602887, GSM1602889, GSM1602890, GSM1602902, GSM1602903, GSM1602908, GSM1602922, GSM1602923, GSM1602924, GSM1602925, GSM1602926, GSM1602927, GSM1602928, GSM1602929, GSM1602931, GSM1602932, GSM1602933, GSM1602934, GSM1602935, GSM1602936, GSM1602937, GSM1602940, GSM1602942, GSM1602945, GSM1602946, GSM1602947, GSM1602953, GSM1602955, GSM1602958, GSM1602967, GSM1602968, GSM1602973, GSM1602974, GSM1602975, GSM1602976, GSM1691861, GSM1691863, GSM1691865, GSM1691868, GSM1691871, GSM1691874, GSM1691877, GSM1691880, GSM1691882, GSM1691886, GSM1691893, GSM1691896, GSM1691897, GSM1691898, GSM1691900, GSM1691901, GSM1691903, GSM1691908, GSM1691909, GSM1691910, GSM1691912, GSM1691916, GSM1691922, GSM1691923, GSM1691925, GSM1691927, GSM1691928, GSM1691929, GSM1691930, GSM1691932, GSM1691933, GSM1691934, GSM1691935, GSM1691937, GSM1691941, GSM1691942, GSM1691943, GSM1691944, GSM1691945, GSM1691949, GSM1691953, GSM1691954, GSM1691958, GSM1691959, GSM1691961, GSM1691963, GSM1691966, GSM1691967, GSM1691968, GSM1691969, GSM1691970, GSM1691972, GSM1691973, GSM1691975, GSM1691977, GSM1691978, GSM1691979, GSM1691980, GSM1691981, GSM1691983, GSM1691984, GSM1691985, GSM1691986, GSM1691987, GSM1691988, GSM1691990, GSM1691992, GSM1691994, GSM1691995, GSM1691996, GSM1691998, GSM1692001, GSM1692002, GSM1692003, GSM1692004, GSM1692006, GSM1692007, GSM1692008, GSM1692009, GSM1692012, GSM1692016, GSM1692017, GSM1692018, GSM1692019, GSM1692021, GSM1692022, GSM1692023, GSM1692024, GSM1692028, GSM1692029, GSM1692030, GSM1692032, GSM1692033, GSM1692037, GSM1692038, GSM1692039, GSM1692040, GSM1692042, GSM1692043, GSM1692044, GSM1692045, GSM1692047, GSM1692050, GSM1692054, GSM1692055, GSM1692056, GSM1692057, GSM1692058, GSM1692059, GSM1692060, GSM1692061, GSM1692063, GSM1692064, GSM1692065, GSM1692070, GSM1692071, GSM1692072, GSM1692073, GSM1692075, GSM1692079, GSM1692081, GSM1692084, GSM1692086, GSM1692088, GSM1692090, GSM1692091, GSM1692092, GSM1692093, GSM1692097, GSM1692098, GSM1692099, GSM1692100, GSM1692103, GSM1692107, GSM1692108, GSM1692110, GSM1692111, GSM1692114, GSM1692115, GSM1692117, GSM1692118, GSM1692119, GSM1692122, GSM1692123, GSM1692124, GSM1692126, GSM1692127, GSM1692128, GSM1692129, GSM1692132, GSM1692135, GSM1692136, GSM1692137, GSM1692138, GSM1692139, GSM1692140, GSM1692141, GSM1692143, GSM1692144, GSM1692146, GSM1692148, GSM1692151, GSM1692153, GSM1692155, GSM1692156, GSM1692157, GSM1692158, GSM1692160, GSM1692165, GSM1692166, GSM1692169, GSM1692170, GSM1692171, GSM1692172, GSM1692174, GSM1692175, GSM1692177, GSM1692178, GSM1692180, GSM1692181, GSM1692183, GSM1692184, GSM1692186, GSM1692188, GSM1692190, GSM1692191, GSM1692194, GSM1692195, GSM1692196, GSM1692198, GSM1692201, GSM1692205, GSM1692206, GSM1692207, GSM1692208, GSM1692211, GSM1692212, GSM1692213, GSM1692214, GSM1692215, GSM1692217, GSM1692219, GSM1692221, GSM1692223, GSM1692224, GSM1692225, GSM1692226, GSM1692228, GSM1692232, GSM1692234, GSM1692235, GSM1692238, GSM1692239, GSM1692240, GSM1692243, GSM1692248, GSM1692249, GSM1692250, GSM1692251, GSM1692254, GSM1692255, GSM1692256, GSM1692258, GSM1692260, GSM1692261, GSM1692267, GSM1692268, GSM1692269, GSM1692271, GSM1692272, GSM1692278, GSM1692279, GSM1692284, GSM1692288, GSM1692289, GSM1692290, GSM1692294, GSM1692295, GSM1692299, GSM1692301, GSM1692302, GSM1692303, GSM1692304, GSM1692305, GSM1692307, GSM1692308, GSM1692309, GSM1692310, GSM1692312, GSM1692313, GSM1692316, GSM1692319, GSM1692322, GSM1692326, GSM1692327, GSM1692329, GSM1692330, GSM1692332, GSM1692335, GSM1692336, GSM1692337, GSM1692339, GSM1692341, GSM1692343, GSM1692347, GSM1692348, GSM1692349, GSM1692350, GSM1692352, GSM1692353, GSM1692354, GSM1692356, GSM1692357, GSM1692358, GSM1692359, GSM1692361, GSM1692362, GSM1692363, GSM1692364, GSM1692365, GSM1692366, GSM1692369, GSM1692374, GSM1692375, GSM1692379, GSM1692381, GSM1692382, GSM1692383, GSM1692384, GSM1692385, GSM1692387, GSM1692388, GSM1692390, GSM1692391, GSM1692393, GSM1692397, GSM1692398, GSM1692399, GSM1692408, GSM1692409, GSM1692410, GSM1692412, GSM1692414, GSM1692416, GSM1692418, GSM1692421, GSM1692424, GSM1692425, GSM1692426, GSM1692428, GSM1692430, GSM1692431, GSM1692434, GSM1692435, GSM1692438, GSM1692440, GSM1692441, GSM1692443, GSM1692444, GSM1692446, GSM1692447, GSM1692448, GSM1692449, GSM1692450, GSM1692453, GSM1692454, GSM1692457, GSM1692458, GSM1692459, GSM1692461, GSM1692463, GSM1692465, GSM1692467, GSM1692471, GSM1692474, GSM1692476, GSM1692477, GSM1692478, GSM1692482, GSM1692484, GSM1692485, GSM1692490, GSM1692494, GSM1692495, GSM1692496, GSM1692499. |
| GSE4607 | C1 | GSM102961, GSM102962, GSM102963, GSM102964, GSM102966, GSM102968, GSM102969, GSM102971, GSM102972, GSM102973, GSM102974, GSM102975, GSM102980, GSM102990, GSM102994, GSM102995, GSM102996, GSM102997, GSM103005, GSM103006, GSM103008, GSM103019, GSM103020, GSM103023, GSM103026, GSM103027, GSM103033, GSM103034, GSM103035, GSM103036, GSM103038, GSM103047, GSM103052, GSM103053, GSM103058, GSM103060, GSM103061, GSM103066, GSM103076, GSM103081, GSM103082, GSM103083. |
|  | C2 | GSM102965, GSM102967, GSM102970, GSM102976, GSM102977, GSM102978, GSM102979, GSM102981, GSM102982, GSM102983, GSM102984, GSM102985, GSM102986, GSM102987, GSM102988, GSM102989, GSM102991, GSM102992, GSM102993, GSM102998, GSM102999, GSM103000, GSM103001, GSM103002, GSM103003, GSM103004, GSM103007, GSM103009, GSM103010, GSM103011, GSM103012, GSM103013, GSM103014, GSM103015, GSM103016, GSM103017, GSM103018, GSM103021, GSM103022, GSM103024, GSM103025, GSM103028, GSM103029, GSM103030, GSM103031, GSM103032, GSM103037, GSM103039, GSM103040, GSM103041, GSM103042, GSM103043, GSM103044, GSM103045, GSM103046, GSM103048, GSM103049, GSM103050, GSM103051, GSM103054, GSM103055, GSM103056, GSM103057, GSM103059, GSM103062, GSM103063, GSM103064, GSM103065, GSM103067, GSM103068, GSM103069, GSM103070, GSM103071, GSM103072, GSM103073, GSM103074, GSM103075, GSM103077, GSM103078, GSM103079, GSM103080. |
| GSE95233 | C1 | GSM2500349, GSM2500350, GSM2500352, GSM2500353, GSM2500354, GSM2500355, GSM2500356, GSM2500357, GSM2500358, GSM2500359, GSM2500360, GSM2500361, GSM2500362, GSM2500363, GSM2500364, GSM2500365, GSM2500366, GSM2500367, GSM2500368, GSM2500369, GSM2500370, GSM2500371, GSM2500372, GSM2500377, GSM2500378, GSM2500380, GSM2500382, GSM2500383, GSM2500384, GSM2500385, GSM2500386, GSM2500387, GSM2500388, GSM2500389, GSM2500393, GSM2500394, GSM2500395, GSM2500396, GSM2500398, GSM2500399, GSM2500400, GSM2500401, GSM2500402, GSM2500406, GSM2500410, GSM2500411, GSM2500412, GSM2500413, GSM2500414, GSM2500415, GSM2500416, GSM2500417, GSM2500418, GSM2500422, GSM2500430, GSM2500431, GSM2500432, GSM2500434, GSM2500435, GSM2500436, GSM2500437, GSM2500439, GSM2500440, GSM2500447, GSM2500448, GSM2500450, GSM2500451, GSM2500455, GSM2500456, GSM2500461, GSM2500462, GSM2500469. |
|  | C2 | GSM2500351, GSM2500373, GSM2500374, GSM2500375, GSM2500376, GSM2500379, GSM2500381, GSM2500390, GSM2500391, GSM2500392, GSM2500397, GSM2500403, GSM2500404, GSM2500405, GSM2500407, GSM2500408, GSM2500409, GSM2500419, GSM2500420, GSM2500421, GSM2500423, GSM2500424, GSM2500425, GSM2500426, GSM2500427, GSM2500428, GSM2500429, GSM2500433, GSM2500438, GSM2500441, GSM2500442, GSM2500443, GSM2500444, GSM2500445, GSM2500446, GSM2500449, GSM2500452, GSM2500453, GSM2500454, GSM2500457, GSM2500458, GSM2500459, GSM2500460, GSM2500463, GSM2500464, GSM2500465, GSM2500466, GSM2500467, GSM2500468, GSM2500470, GSM2500471, GSM2500472. |
