# Supplementary material for: Unlocking the potential of senescence-related gene signature as a diagnostic and prognostic biomarker in sepsis: insights from meta-analyses, single-cell RNA sequencing, and in vitro experiments
Source: Aging (Albany NY). 2024 Feb 26;16(4):3989–4013. doi: 10.18632/aging.205574 (PMC10929830; doi:10.18632/aging.205574)
Supplement: Supplementary Table 8 [file aging-16-205574-s008.pdf]

**Supplementary Table 8. The AUCs indicated the diagnostic ability of TGFBI, MAD1L1, and SRS in each cohort.**

| Cohort | AUCs | | |
|---|---|---|---|
| | TGFBI | MAD1L1 | SRS |
| GSE4607 | 0.877 | 0.766 | 0.853 |
| GSE9692 | 0.873 | 0.773 | 0.889 |
| GSE13904 | 0.811 | 0.694 | 0.784 |
| GSE26378 | 0.821 | 0.687 | 0.810 |
| GSE26440 | 0.830 | 0.775 | 0.839 |
| GSE28750 | 0.905 | 0.995 | 0.893 |
| GSE54514 | 0.484 | 0.571 | 0.508 |
| GSE57065 | 0.927 | 0.869 | 0.950 |
| GSE65682 | 0.874 | 0.948 | 0.962 |
| GSE67652 | 0.510 | 0.521 | 0.507 |
| GSE69063 | 0.808 | 0.920 | 0.938 |
| GSE69528 | 0.806 | 0.820 | 0.870 |
| GSE95233 | 0.919 | 0.761 | 0.928 |
| GSE131761 | 0.964 | 0.879 | 0.962 |
